# Acupuncture in the treatment of chemotherapy-induced peripheral neuropathy: a meta-analysis and data mining

**DOI:** 10.3389/fneur.2024.1442841

**Published:** 2024-10-29

**Authors:** Limeng Li, Yingxue Huang, Chengfei An, Ning Jing, Chuhan Xu, Xiaoyu Wang, Huanan Li, Tao Tan

**Affiliations:** ^1^Tuina Department, First Teaching Hospital of Tianjin University of Traditional Chinese Medicine, Tianjin, China; ^2^National Administration of Traditional Chinese Medicine Level Three Laboratory for Tuina Technique Biological Effects, Tianjin, China

**Keywords:** acupuncture, systematic review, chemotherapy-induced peripheral neuropathy, data mining, complementary and alternative therapies

## Abstract

**Background:**

The efficacy and acupoint selection of acupuncture in treating chemotherapy-induced peripheral neuropathy (CIPN) remain controversial. This study aims to explore the specific efficacy and acupoint selection of acupuncture in treating CIPN through a meta-analysis and data mining.

**Methods:**

Searching for clinical trials on acupuncture treatment for CIPN in 8 databases, evaluating its efficacy and safety through a meta-analysis, and exploring its acupoint selection through data mining.

**Results:**

The meta-analysis included 21 studies and 2,121 patients, showing that compared with the control group, the acupuncture group could significantly improve neuropathic pain intensity (SMD = −0.66, 95% CI [−1.07, −0.25], *p* = 0.002), significantly reduce the NCI-CTCAE (MD = −0.29, 95%CI [−0.50, −0.08], *p* < 0.01), significantly reduce the FACT-NXT score (MD = 2.09, 95% CI [0.73,3.45], *p* < 0.05), significantly increase the motor conduction velocities (MCV) of median nerve (MD = 2.38, 95% CI [2.10, 2.67], *p* < 0.001), the sensory conduction velocities (SCV) of the median nerve (MD = 0.56, 95 %CI [−1.45, 2.57], *p* = 0.58), the SCV of the tibial nerve (MD = 1.78, 95% CI [0.50, 3.05], *p* < 0.01), and the SCV of sural nerves (MD = 4.60, 95% CI [0.17, 9.02], *p* < 0.05), as well as improving the quality of life score (MD =7.35, 95% CI [1.53, 13.18], *p* = 0.01). Data mining showed that the core acupoints for acupuncture treatment of CIPN were LI4, ST36, LI11, LR3, and SP6.

**Conclusion:**

Acupuncture can improve the neuropathic pain intensity, the intensity of the CIPN, MCV of the median nerve, SCV of the tibial nerve and peroneal nerve, quality of life, and has good safety in CIPN patients. LI4 (Hegu), ST36 (Zusanli), LI11 (Quchi), LR3 (Taichong), and SP6 (Sanyinjiao) are the core acupuncture points for treating CIPN, and this protocol has the potential to become a supplementary treatment for CIPN.

**Systematic review registration:**

https://www.crd.york.ac.uk/prospero, identifier CRD42024551137.

## 1 Introduction

Cancer remains the leading cause of death worldwide, with approximately 16.5 million cancer survivors in the United States in 2016 and a projected number of nearly 26.1 million by 2040 (1). Despite the remarkable clinical results of chemotherapy in cancer treatment, numerous patients who undergo chemotherapy experience chronic pain that severely affects their quality of life. Chemotherapy-induced peripheral neuropathy (CIPN) is a common complication after treatment with various commonly used anticancer drugs (2), such as platinum drugs, taxanes, vincristine drugs, and proteasome inhibitors can induce CIPN, which is one of the most complex symptoms in the clinical treatment of cancer patients (3). A meta-analysis of 31 studies, 4,179 cancer patients, found that the incidence was 68.1 and 60%, respectively, at 1 and 3 months after the start of chemotherapy, and the incidence remained at 30.0% after 6 months (4). Patients experience a range of symptoms and signs, including paresthesia, such as tingling, pain, numbness, or burning. Motor dysfunction: muscle weakness or impaired coordination that may cause difficulty walking or a reduced ability to hold objects. Autonomic dysfunction: May cause abnormal sweating, fluctuating blood pressure, digestive problems, or difficulty urinating (5, 6). These symptoms affect the patients’ sensory function, motor abilities, quality of sleep, psychological status and other aspects, and seriously reduce the quality of life for patients (7, 8). There are very limited available and effective clinical treatment strategies for CIPN. And to relieve the symptoms, occasionally the drug dose has to be reduced or temporarily stopped, which undoubtedly constitutes a great limitation to the treatment of cancer (9).

Acupuncture, as a kind of traditional Chinese medicine therapy, can stimulate the body surface acupoints, give play to the proximal treatment, remote treatment, bidirectional regulation, special functions of acupoints, and adjust the functions of qi, blood and, zang-fu organs through the whole-body meridian conduction, to achieve the purpose of preventing and treating diseases. Acupuncture treatment may alleviate the neurotoxicity of CIPN by down-regulating the level of Na + channels, promoting the secretion of neurotransmitters such as glutamate, inhibiting oxidative stress, and promoting the release of anti-inflammatory cytokines.

However, the efficacy and point selection of acupuncture in the treatment of CIPN remain controversial (10), due to the lack of large-scale the clinical trials, and conflicting results have been reported in relevant randomized controlled trials. Recently, several high-level clinical studies were published. At present, randomized controlled trials of acupuncture intervention timing, course of treatment, intervention type, and treatment frequency are different, resulting in poor quality of evidence on the use of acupuncture for clinical treatment of patients with CIPN. As a new method of objective analysis based on statistical principles, meta-analysis has received more and more attention and application (11–14). This method can comprehensively and objectively analyze the results of multiple independent studies with the same research purpose and obtain comprehensive conclusions. Therefore, this study will systematically evaluate the intervention effect of acupuncture on CIPN. This review differs from previous reviews in that it considers a wider range of outcome measures and analyses the effects of acupuncture intervention variables. At the time, explore the core acupoints of acupuncture on CIPN by using data mining technology, in order to provide reliable data for clinical applications.

## 2 Materials and methods

The present study was conducted by the 2020 Statement on Preferred Reporting Items for Systematic Reviews and Meta-analyses (PRISMA 2020). This systematic review and meta-analysis has been registered with the PROSPERO Registry (CRD42022370952).

### 2.1 Meta-analysis methods

#### 2.1.1 Literature search

The two researchers independently searched Pubmed, Cochrane Library, Web of Science, Embase, China National Knowledge Infrastructure (CNKI), Wanfang Database, Weipu (VIP) Database, Sinomed (CBM), self-built repositories - published articles in January 2024. In addition, references to clinical trial registries and websites, as well as relevant reviews and systematic reviews, were searched for all eligible studies. The search strategy is presented in the Supplementary material 1.

#### 2.1.2 Inclusion criteria

(1) The study type was a randomized controlled clinical trial. (2) Participants were cancer patients with or without CPNI, regardless of cancer type or stage. (3) The intervention group was acupuncture/electroacupuncture or acupuncture/electroacupuncture combined with conventional rehabilitation/western medicine therapy. (4) The control group was treated with sham acupuncture or conventional rehabilitation or other Western medicine. (5) Primary outcome measures include any related measures of CIPN.

#### 2.1.3 Exclusion criteria

(1) Studies comparing different types of acupuncture. (2) Studies with a total sample size of less than 10. (3) Conference abstracts, non-randomized controlled trials, reviews, systematic reviews, qualitative studies, protocols, case reports, etc.

#### 2.1.4 Literature selection and data extraction

Two researchers (LLM and HYX) independently conducted literature screening and data extraction. Using Endnote 20.1 software, they removed duplicate articles. Following that, they screened out studies that did not meet the criteria by reading the titles and abstracts. Finally, they read the full texts of the remaining articles, determined the final selection based on the inclusion and exclusion criteria, and then extracted data from randomized controlled trials. First author, publication year, study subjects, study design (randomized, blind), intervention measures, intervention period, evaluation measures, outcomes, and adverse events were included. Subgroups were split if there were three - or multi-arm studies. If data is missing, the author will be contacted via email for further information. Any disagreement is reviewed by a third reviewer (TT) and resolved through discussion by all reviewers.

#### 2.1.5 Risk of bias assessment

The risk of bias in the included studies was assessed by two investigators (LLM and ACF) using the Cochrane Manual of Systematic Review 5.1.0 RCT Bias Risk Assessment tool, encompassing seven aspects: random sequence generation, allocation concealment, blinding of patients and investigators, blinding of outcome evaluators, incomplete outcome data reporting, selective reporting, and other sources of bias. In cases where there was disagreement between the two evaluators, a third evaluator (LHN) was involved to resolve any discrepancies.

#### 2.1.6 Statistical analysis

The meta-analysis was performed using Revman 5.3 software. Means and standard deviations of pretreatment and posttreatment results were collected for each group. Multiple levels of data are combined as continuous variables. Continuity variables were expressed in weighted mean difference (MD) or standardized mean difference (SMD), and 95% confidence intervals (CI) were calculated. Referring to the study by Chen HT, for studies with two or more interventions/controls, participants were divided into different subgroups for assessment to prevent sample size overlap (15). The heterogeneity among the included studies was analyzed by χ^2^ test (*α* = 0.05), and the heterogeneity was quantitatively determined by *I^2^*. When *I^2^* = 0%, no heterogeneity was considered, and the fixed effect model was used to combine the data. When *I*^2^ was less than 50%, the heterogeneity was not considered significant, and the random effects model was conservatively used to combine the data. When *I^2^* ≥ 50%, the heterogeneity is considered significant, and the random effects model is used to combine the data. Sources of heterogeneity were analyzed as far as possible, and obvious clinical heterogeneity was treated by sensitivity analysis or descriptive analysis (11, 12, 16). If more than 10 studies were included, the Begg’s tests and funnel plot were analyzed using Stata 17 software. If the funnel plot is symmetrical and *p* > 0.05, then the possibility of publication bias is relatively low.

### 2.2 Data mining methods

#### 2.2.1 Literature search

The literature retrieval strategy is the same as meta-analysis.

#### 2.2.2 Inclusion criteria

(1) Randomized controlled trials or case–control studies. (2) The included subjects were patients with CIPN. (3) The experimental group was treated with acupuncture, and the acupoint records were complete. (4)The efficacy of acupuncture was definite.

#### 2.2.3 Exclusion criteria

(1) Duplicate publications. (2) Literature on non-acupuncture therapy. (3)The full study was not available.

#### 2.2.4 Standardization of acupoint names

The name of acupoints was standardized according to the name and Location of acupoints. For example, “Zusanli” is standardized as “ST36” and “Bafeng” is standardized as “EX-LE10.”

#### 2.2.5 Data analysis

The Excel 2021 software was used to calculate the frequency of acupoints. R4.3.1 software was used to analyze association rules. Association rules analysis using arules software package, Apriori algorithm, set minimum support of 0.3, minimum confidence of 0.6, lift >1, and use arules *Viz* software package for visualization.

## 3 Results

### 3.1 Literature screening

**Figure 1:**
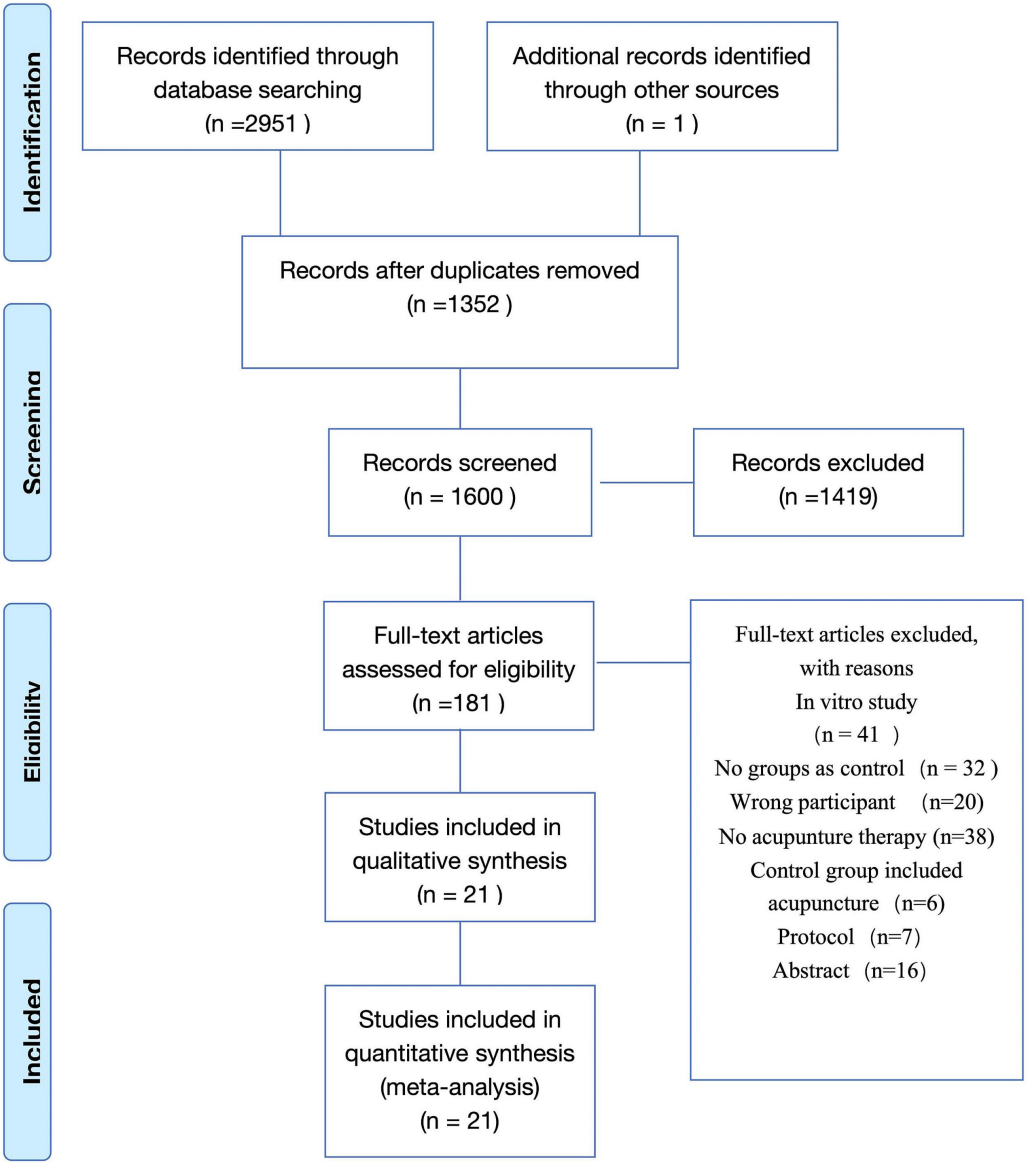
PRISMA flow chart.

The article screening flow chart is in Figure 1. A total of 2,952 literature were searched, among which 1,352 duplicate literature were excluded, 1,419 irrelevant literature were excluded by title and abstract, 160 literature were excluded by full-text review, and 21 studies (10, 17–36) were finally included (Figure 1).

### 3.2 Basic characteristics of included studies

A total of 21 clinical studies were included, of which 3 were published in Chinese and 18 in English. The studies were published between 2013 and 2023, with 17 (80.95%) published in the last five years. Of the included studies, 10 studies were conducted in China, 5 studies in the United States, 1 study in collaboration with institutions in China and Iran, 1 in Brazil, 1 in Sweden, and 1 in Germany. A total of 2,121 subjects were included, including 1,048 in the experimental group and 1,073 in the control group. Baseline information such as sex, age, cancer type, and chemotherapy regimen were comparable between the experimental and control groups. The basic characteristics of the included studies are shown in Table 1.

**Table 1:** Basic characteristics of the included studies.

Author, year		Sample size	Age	Intervention	Cancer type	Frequency (weeks/days)	Intervention Duration	Chemotherapy	Existing CIPN	Outcome measure
Zhi, W. I., 2023 (17)	Intervention	23	60.2 (36.3–82.1)	AC	Solid tumor	\	Duration:8wks	\	Yes	TDT, VDT, THDT, THT
	Control^1^	22	62.3 (44.5–86.0)	Sham-AC						
	Control^2^	18	58.5 (48.2–71.7)	UC						
Lagerstedt, K., 2023 (18)	Intervention	6	68 (63–76)	AC	CRC	2 times per week	Duration:5wks	Oxaliplatin	Yes	EOR-QLQC30, VAS
	Control	6	69 (64–70)	Sham-AC						
Huang, M. C., 2023 (19)	Intervention	13	52.0 (36.5, 53.0)	AC	CA	1 time in 2 weeks	duration of CTX (The majority had 8 or more cycles of chemotherapy)	Oxaliplatin	NO	NCV, FACT-G, FACT-Nxt, BPI-SF
	Control	13	52.0 (40.0, 57.0)	Sham-AC						
Chan, K. Y. 2023 (20)	Intervention	27	60.0 ± 8.57	EA	CRC	1 time per week	Duration:12wks	Oxaliplatin	NO	FACT-Nxt, NRS, EOR-QLQC30
	Control	28	62.5 ± 7.62	Sham-EA						
Wang, L., 2023 (21)	Intervention	26	70	AC	MM	1 time per week	Duration:2wks	\	Yes	NCI-CTC
	Control	28	68	Vitamin B						
Zhang, C., 2022 (22)	Intervention	60	48.11 ± 6.21	AC+ Vitamin B	\	1 time per week	Duration:2wks	GEM	Yes	NCI-CTC, NCV
	Control	60		Vitamin B						
Liu, Y. Q, 2022 (23)	Intervention^a^	15	48.24 ± 6.31	AC+ Met	gastric carcinoma	1 time per week	Duration:3 days	\	No	NCI-CTC
	Intervention^b^	15		AC						
	Control			Met						
Stringer, J., 2022 (24)	Intervention	53	61 (37–76)	AC + SC	\	1 time per week	Duration:10wks	\	Yes	MYMOP2, NCI-CTC, EOR-QLQC30, EORTC QLQ-CIPN20, likert pain scale
	Control	56	60 (29–79)	SC						
Friedemann, T., 2022 (25)	Intervention	27	57.68 (41–82)	AC+ recovery	\	1 time per week	Duration:10wks	\	Yes	NCS, PROM, NRS
	Control	28		recovery						
BEN-Arye, E., 2022 (26)	Intervention^a^	69	57.9 ± 12.1	AC	\	2 times per week	Duration:6wks	Tax	Yes	FACT-TAX, QLQ-C30, MYCAW
	Intervention^b^	32	59.9 ± 10.9	AC + CIM						
	Control		55.8 ± 13.1	SC						
Huang, C. C., 2022 (10)	Intervention	10	47.1 ± 11.07	AC	\	2 treatments per week for the first 6 weeks, then 1 treatment per week for the next 3 weeks	Duration:9wks	\	Yes	vof, BPI-SF, FACT-Nxt, WHOQOL-BREF
	Control	10	52.1 ± 11.18	Sham-AC						
Ting Bao 2021 (27)	Intervention	27	59.7 (36.3–85.9)	EA	\	biweekly treatments for the first 2 weeks and weekly treatments thereafter.	Duration:8wks	\	Yes	FACT-Ntx, HADS, ISI, BFI
	Control^1^	24	60.3 (51.0–79.9)	Sham-EA						
	Control^2^	24	62.7 (43–86)	SC						
Lu, W. D., 2020 (28)	Intervention	14	54 (32–68)	AC + EA	\	\	Duration:8wks	Taxanes	Yes	PNQ, FACT-Nxt, BFI-SF, QLQ-C30
	Control	17	53 (26–71)	Wait for treatment						
Iravani, S., 2020 (29)	Intervention	19	57.95 ± 10.39	AC	\	3 times per week	Duration:4wks	\	Yes	NRS (symptom severity in CIPN), NCI-CTC, NCS
	Control	19	58.79 ± 8.36	B1 + Babapentin						
Ting Bao 2020 (30)	Intervention	24	59.7 (36.3–85.9)	EA	\	biweekly treatments for the first 2 weeks and weekly treatments thereafter.	Duration:8wks	\	Yes	NRS
	Control^1^	23		Sham-EA						
D’Alessandro, E. G., 2022 (31)	Intervention	15	57.68 (41–82)	AC + SC	\	2 times per week	Duration:5wks	\	Yes	NCI-CTC, FIM, QLQ-C30, VAS
	Control	14		SC						
Molassiotis, A., 2019 (32)	Intervention	43	57.1 ± 7.7	AC	\	2 times per week	Duration:8wks	\	Yes	FACT-Nxt, FACT-G, BPI-SF
	Control	43		SC						
Sui, M. H.2018 (33)	Intervention^a^	10	59.1 ± 7.62	EA	\	3 times per week	Duration:3wks	\	Yes	VAS, KPS
	Intervention^b^	10	57.2 ± 8.48	EA + Met						
	Control		57.7 ± 8.33	Met						
Han, X. Y., 2017 (34)	Intervention	49	62.46	AC+ Met	\	The acupunctures were done daily for 3 days, then once every alternate day for 10 days as a treatment cycle.	Duration:3 CTX cycles (10 days as a treatment cycle. Each cycle was repeated every 28 days and the complete treatment included three cycles)	\	Yes	NSV, NTX-11, NCI-CTC
	Control	49	65.29	Met						
Greenlee, H., 2016 (35)	Intervention	25	51.8 ± 10.7	EA	\	1 time per week	Duration:12wks	Taxanes	No	FACT-Nxt, BPI-SF, NRS, FACT-Tax
	Control	23	51.8 ± 10.7	Sham-EA						
Rostock, M.2013 (36)	Intervention	13	49.9 ± 9.6	EA	\	\	Duration:3wks	\	Yes	NRS (symptom severity), QLQ-C30, NCI-CTC
	Control		56.3 ± 11.1	vitamin B1and B6						

AC, Acupuncture; UC, Usual care; TDT, Tactile detection threshold; VDT, Vibration detection threshold; THDT, cool and warm thermal detection threshold; THT, cold and hot pain thermal threshold; CRC, Colorectal cancer; EORTC QLQ-C30, European Organization for Research and Treatment of Cancer Quality of Life Questionnaire; NCI-CTCAE, National Cancer Institute Common Terminology Criteria for Adverse Events; NCV, Neural conduction velocity; FACT-G, functional assessment of cancer therapy/gynecologic oncology group-neurotoxicity-general; FACT/NTx, Functional Assessment of Cancer Therapy/Neurotoxicity Questionnaire; BPI-SF, *Brief Pain Inventory Short Form*; EA, electroacupuncture; MM, Multiple myeloma; BC, Breast cancer; ME, mecobalamin; CIM; BFI, Brief Fatigue Inventory; HADS, Hospital Anxiety and Depression Scale; ISI, the insomnia severity index; PNQ, patient neurotoxicity questionnaire; SC, Standard care; *PROM*, Patient-Reported Outcomes Measure; FACT-TAX, Functional Assessment of Cancer Therapy Taxane scale.

^a^Triple-arm study design, intervention group 1.

^b^Triple-arm study design, intervention group 2.

^1^Triple-arm study design, control group 1.

^2^Triple-arm study design, control group 2.

### 3.3 Risk of bias assessment

**Figure 2:**
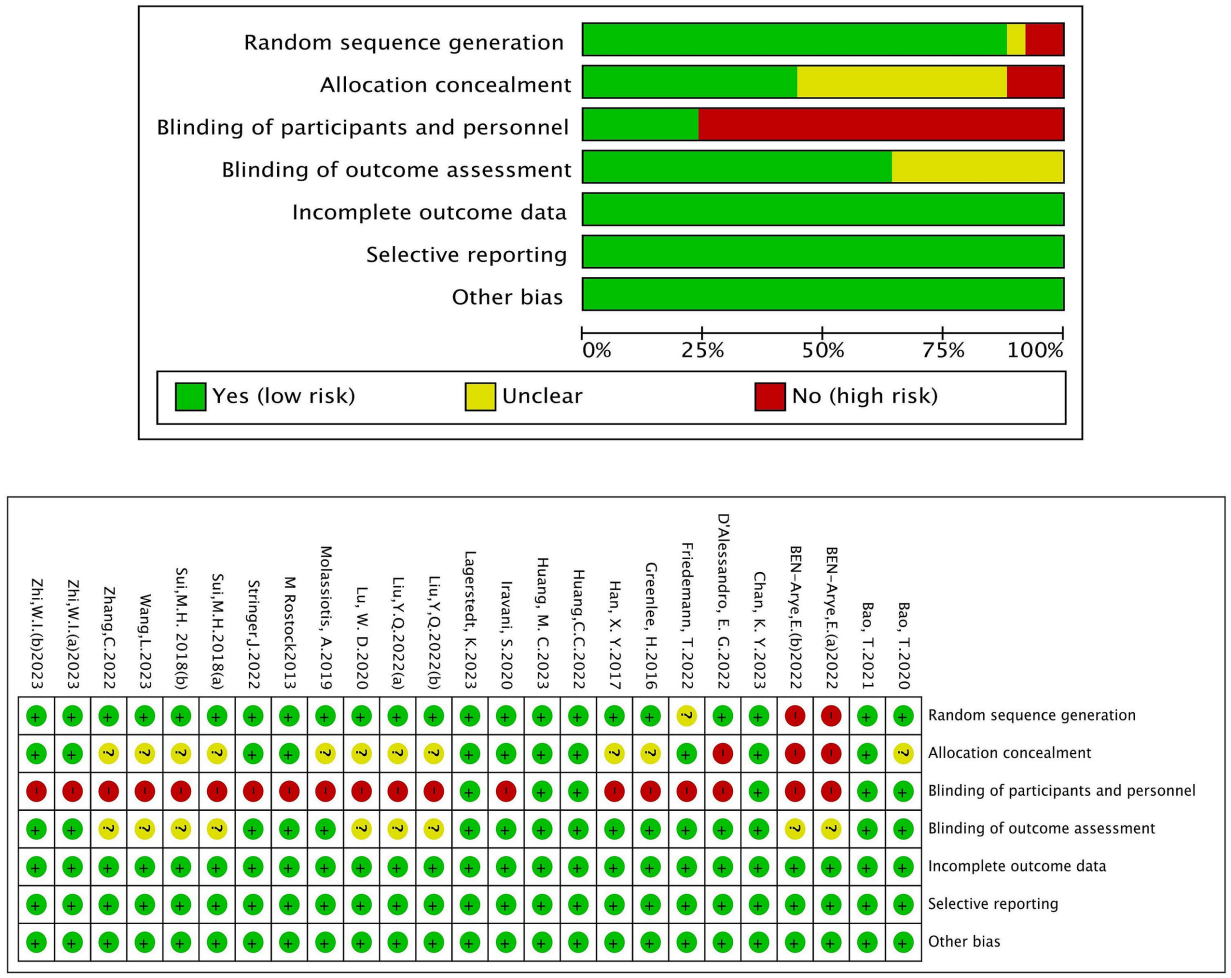
Risk of bias graph.

The risk of random method bias was not clear in 1 study, and 11 studies describing envelopes and other ways to assign hiding were rated as low risk. Due to the special nature of acupuncture, 14 studies did not implement blind interventions for patients and participants rated as high risk. The risk of other bias was low. The details are shown in Figure 2.

### 3.4 Meta-analysis results

#### 3.4.1 Pain intensity

**Figure 3:**
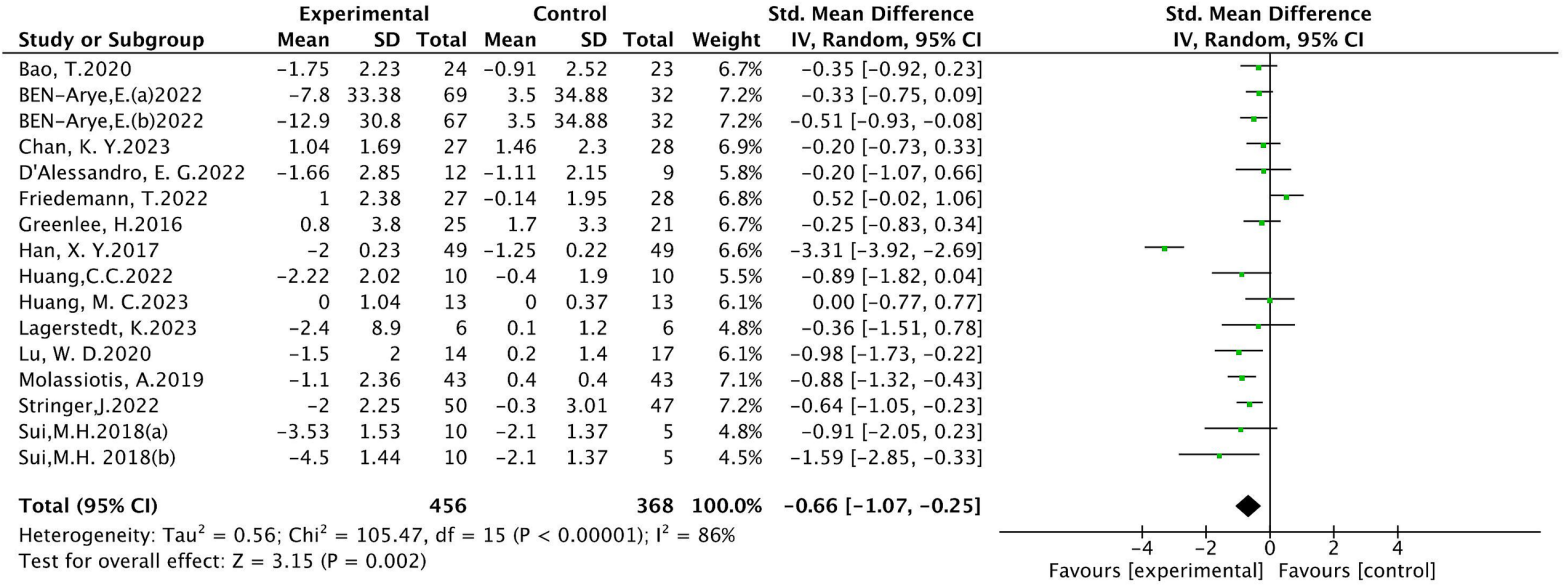
Meta analysis of pain intensity.

**Figure 4:**
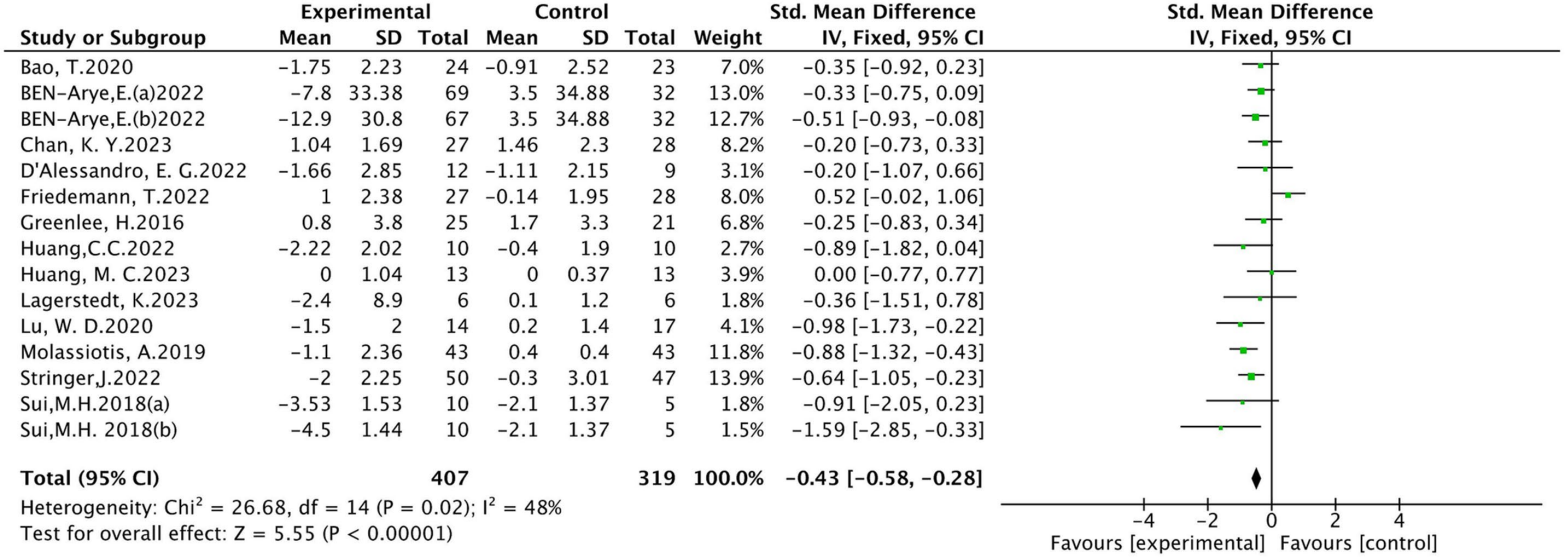
Meta analysis of pain intensity (after removing 1 study).

Sixteen (10, 18–20, 24–26, 28, 30–35) studies evaluated peripheral neuropathic pain intensity involving a total of 824 patients. Of these, 4 studies (18, 31, 33, 34) were evaluated using the visual analogue scale (VAS), 5 studies (10, 24, 28, 32, 35) using BPI-SF, and 4 studies (19, 20, 25, 30) using the Numeric Rating Scale (NRS), 1 study (26) using EORTC QLQ-30-Pain scale, The results of heterogeneity analysis show *p* < 0.001 and *I*^2^ = 86%, indicating a high heterogeneity, so a random effects model was used for the analysis. The meta-analysis results showed that compared with the control group, the pain intensity in the acupuncture group decreased more (SMD = −0.66, 95% CI [−1.07, −0.25], *p* = 0.002), illustrated in Figure 3. Sensitivity analysis showed that after removal of a study (34), *I*^2^ decreased to 48% (SMD = −0.43, 95% CI [−0.58, −0.28], *p* < 0.001) (see Figure 4). The intervention period of this study was greatly larger than that of other studies (3 chemotherapy cycles), suggesting that this study may be the source of high heterogeneity, and the length of acupuncture intervention affects the difference in acupuncture treatment results.

#### 3.4.2 NCI-CTCAE

**Figure 5:**
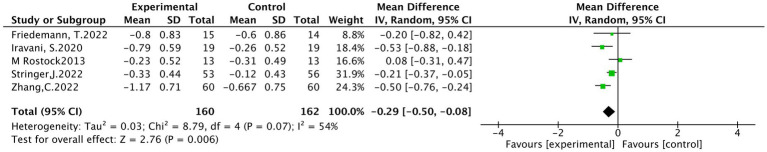
Meta analysis of NCI-CTCAE.

It into five studies (22, 24, 25, 29, 36), involving 322 patients, 160 in the treatment group and 162 people in the control group. A random effects model was used for analysis. Meta-analysis showed a statistically significant difference in the grade of peripheral neurotoxicity between the treatment and control groups (MD = −0.29, 95% CI [−0.50, −0.08], *p* < 0.001), as shown in Figure 5.

#### 3.4.3 FACT-NXT

**Figure 6:**
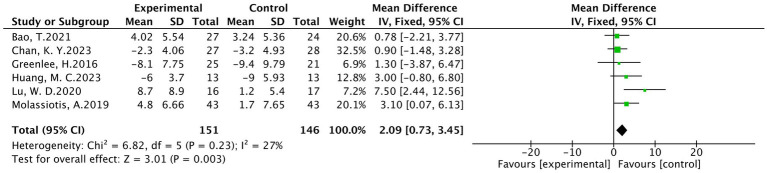
Meta analysis of FACT-NXT.

A total of six studies (19, 20, 27, 28, 32, 35) were included, involving 297 patients, of which 151 were in the treatment group and 146 were in the control group. The results of heterogeneity analysis show *p* = 0.23 and *I*^2^ = 27%. Therefore, a fixed effects model was used for analysis. Meta-analysis showed statistically significant between the treatment and control groups (MD = 2.09, 95% CI [0.73,3.45], *p* < 0.05), as detailed in Figure 6.

#### 3.4.4 Neural conduction velocity

(1) Motor conduction velocities (MCV) of median nerves

**Figure 7:**
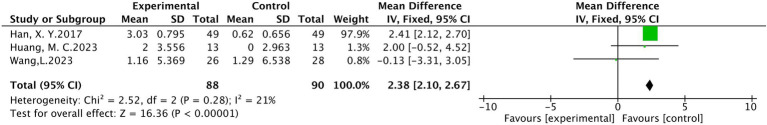
Meta analysis of MCV of median nerve.

In total, three studies were included, involving 178 patients (19, 21, 34), with 88 patients in the treatment group and 90 in the control group. The results of heterogeneity analysis show *p* = 0.28 and *I*^2^ = 21%, indicating homogeneity among the statistical variables. A fixed effects model analysis showed that there was a statistically significant difference in the motor conduction velocity of the median nerve between the treatment group and the control group (MD = 2.38, 95% CI [2.10, 2.67], *p* < 0.001), illustrated in Figure 7.

(2) Sensory conduction velocities (SCV) of the median nerve

**Figure 8:**
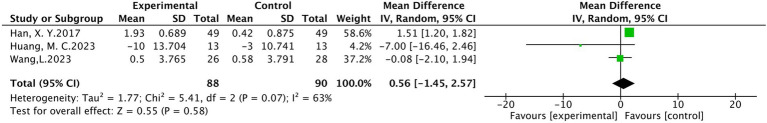
Meta analysis of SCV of median nerve.

There were three studies included in the analysis, involving a total of 178 patients (19, 21, 34), of which 88 were in the treatment group and 90 were in the control group. The results of heterogeneity analysis show *p* = 0.07 and *I*^2^ = 63%, indicating a high heterogeneity. The random effects model analysis showed no statistically significant difference between the treatment group and the control group in median nerve conduction velocity (MD = 0.56, 95 %CI [−1.45, 2.57], *p* = 0.58), as detailed in Figure 8.

(3) SCV of the tibial nerve

**Figure 9:**
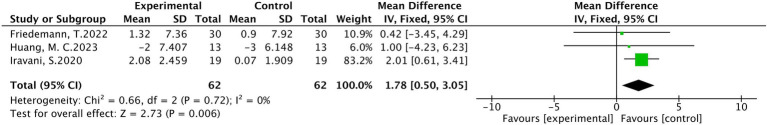
Meta analysis of SCV of tibial nerve.

In a total of three studies involving 124 patients (19, 25, 29), with 62 patients in the treatment group and 62 in the control group. The results of heterogeneity analysis show *p* = 0.72 and *I*^2^ = 0%, indicating homogeneity among various statistics. A fixed effects model analysis showed that there was a statistically significant difference in the median nerve motor conduction velocity between the treatment group and the control group (MD = 1.78, 95% CI [0.50, 3.05], *p* < 0.01), These findings are presented in Figure 9.

(4) SCV of the sural nerver

**Figure 10:**
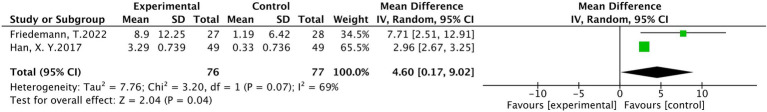
Meta analysis of SCV of the sural never.

A total of 2 studies were included (25, 34) involving 153 patients, with 76 in the treatment group and 77 in the control group. The results of heterogeneity analysis show *p* = 0.07 and *I*^2^ = 69%, indicating a high heterogeneity. The random effects model analysis showed that there was a statistically significant difference in the median nerve motor conduction velocity between the treatment group and the control group (MD = 4.60, 95% CI [0.17, 9.02], *p* < 0.05), as shown in Figure 10.

#### 3.4.5 Quality of life score (QOL)

**Figure 11:**
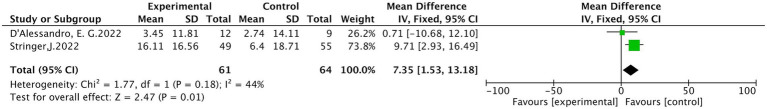
Meta-analysis of total quality of life score.

Two studies (24, 31) reported the total score of the EORTC QLQ-30. The results of heterogeneity analysis show *p* = 0.18 and *I*^2^ = 44%, indicating moderate heterogeneity. The fixed effects model analysis showed a statistically significant difference in scores between the treatment and control groups (MD =7.35, 95% CI [1.53, 13.18], *p* = 0.01), detailed in Figure 11.

#### 3.4.6 Other indicators not combined

**Figure 12:**
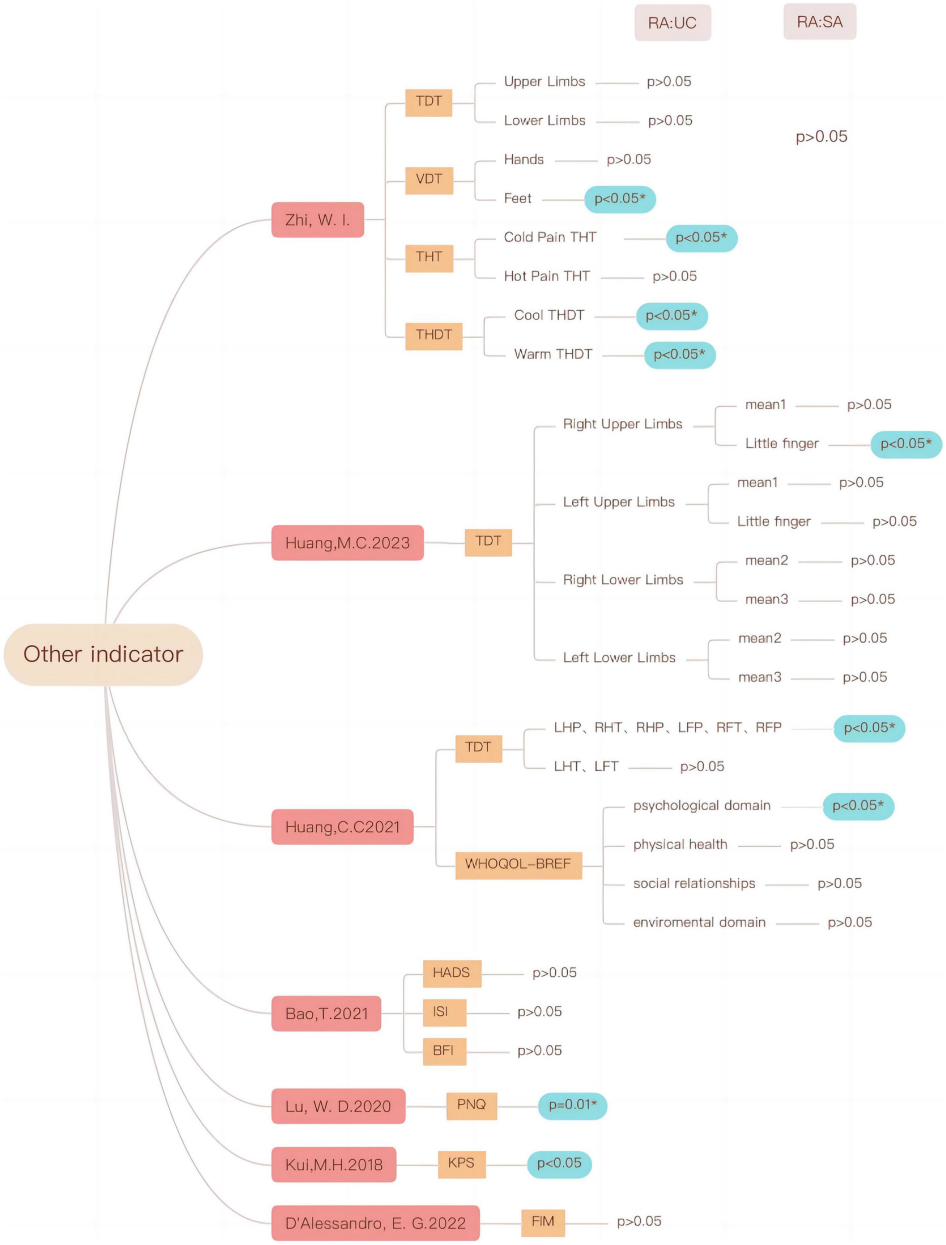
Effects of acupuncture on other indicators in CIPN patients.

Due to the variety of CIPN indicators, there are both subjective and objective indicators, and individual indicators are reported less frequently or difficult to combine, for example, the results of different vibration sensing detection locations are very different. Therefore additional indicators are summarized. The results showed that acupuncture in patients with CIPN may be at the threshold of vibration sensation, cool and warm thermal detection threshold (THDT), cold and hot pain thermal threshold, the quality of life, and so on (see Figure 12).

#### 3.4.7 Subgroup analysis

In subgroup analysis, the pain intensity was used as the outcome, and the influence of acupuncture intervention time, type of intervention, treatment duration, and weekly intervention frequency were discussed. The results showed that prophylactic acupuncture intervention did not effectively reduce pain intensity before CIPN (SMD = −0.11, 95%CI −1.46 to 0.24, *p* = 0.53). Acupuncture after the onset of CIPN can effectively reduce pain intensity (SMD = −0.80, 95% CI −1.34 to −0.26, *p* < 0.05). Subgroup analysis according to intervention type showed that electropuncture (SMD = −0.42, 95%CI −0.77 to −0.06, *p* = 0.02), hand acupuncture (SMD = −0.71, 95%CI −1.35 to −0.08, *p* = 0.03) and electropuncture combined plus hand acupuncture (SMD = −0.98, 95%CI −1.73 to −0.22, *p* = 0.01) were superior to control group in improving pain intensity. Judging from the course of treatment, within 6 weeks of acupuncture (SMD = −0.64, 95%CI −1.22 to −0.05, *p* < 0.05), within 6 to 12 weeks of acupuncture (SMD = −0.38, 95% CI 0.64 to −0.13, *p* < 0.01) can effectively reduce pain intensity. However, there was no significant difference in pain intensity in patients with an intervention period greater than 12 weeks (*p* = 0.31). From the analysis of weekly intervention frequency, acupuncture twice (SMD = −0.53, 95% CI −0.76 to −0.29, *p* < 0.001) or three times a week (SMD = −1.21, 95% CI −2.06 to −0.37, *p* < 0.01) can effectively reduce the pain intensity. However, acupuncture once a week does not show statistically significant compared with the control group (SMD = −0.18, 95% CI −0.43 to 0.07, *p* = 0.15) (see Table 2).

**Table 2:** Subgroup analysis.

Subject	Subgroup	Number of trials	Number of patients	SMD (95%CI)	*p*-value	*I* ^2^
Existing CIPN	No	2	81	−0.14 [−0.58, 0.30]	0.53	0
	Yes	14	743	−0.74 [−1.20, −0.28]	0.002	87
Type of intervention	Electroacupuncture	5	178	−0.42 [−0.77, −0.06]	0.02	20
	Manual acupuncture	8	594	−0.71 [−1.35, −0.08]	0.03	92
	Manual acupuncture plus Electroacupuncture	1	31	−0.98 [−1.73, −0.22]	0.01	/
Intervention cycle	≤6 weeks	6	298	−0.47 [−0.73, −0.21]	0.001	0
	6 ~ 12 weeks	0	389	−0.47 [−0.86, −0.07]	0.02	0
	≥12 weeks	3	179	−1.17 [−3.29, 0.95]	0.28	97
Intervention frequency	1 times per week	4	253	−0.18 [−0.43, 0.07]	0.15	74
	2 times per week	6	366	−0.53 [−0.76, −0.29]	0.001	0
	3 times per week	2	30	−1.21 [−2.06, −0.37]	0.005	0

#### 3.4.8 Safety endpoint

Five studies (20, 23–25, 29) have reported adverse reactions associated with acupuncture, which mainly included blood clotting, pain, and needle stagnation, among others. They returned to normal after treatment, and no serious adverse reactions were reported.

#### 3.4.9 Publication bias

**Figure 13:**
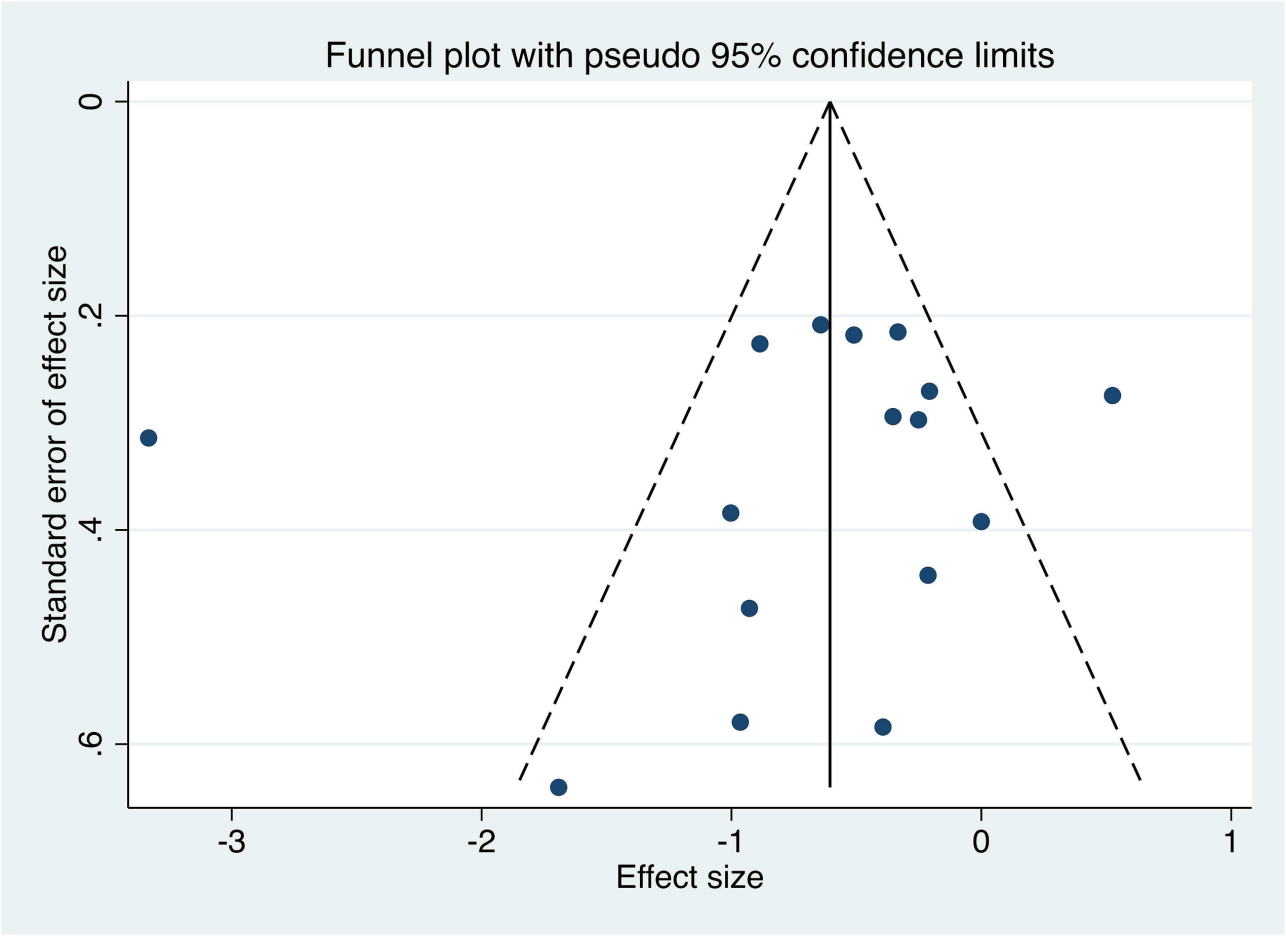
Publication bias.

There are 16 studies on pain intensity as an indicator of therapeutic effect. Thus the funnel plot analysis with pain intensity as an example shows that the funnel plot for comparison of pain intensity between the two groups is roughly symmetrical and the Egger’s test *p* > 0.05, indicating that there is no significant risk of publication bias. These findings are presented in Figure 13.

### 3.5 Data mining results

#### 3.5.1 Literature screening

A total of 2,952 studies were searched. According to inclusion and exclusion criteria, 59 clinical studies were included. A total of 77 acupoints were involved, with a total frequency of 826.

#### 3.5.2 Frequency analysis

Frequency analysis was performed on the acupoints, resulting in 17 common acupoints with frequencies greater than 5, namely Hegu (LI4), Zusanli (ST36), Quchi (LI11), Taichong (LR3), Sanyinjiao (SP6), Bafeng (EX-LE10), Waiguan (TE5), Baxie (EX-UE9), Qihai (CV6), Yanglingquan (GB34), Neiguan (PC6), Xuehai (SP10), Yinlingquan (SP9), Guanyuan (CV4), Daling (PC7), Houxi (SI3), Jiexi (ST41), as shown in Table 3.

**Table 3:** Common acupoints with a frequency greater than 5.

Rank	Acupoint	Frequency (*n*/%)	Location
1	LI4	46 (77.97)	On the dorsum of the hand, between the 1nd and 2nd metacarpal bones, at the midpoint of the 2nd metacarpal bone on the radial side.
2	ST36	45 (76.27)	On the anterior aspect of the lower leg, 3 cun below ST 35, one finger-breadth from the anterior crest of the tibia.
3	LI11	37 (62.71)	On the extensor aspect of the thumb, at the junction of lines drawn along the radial border of the nail and the base of the nail, approximately 0.1 cun from the corner of the nail.
4	LR3	30 (50.85)	Located on the dorsum of the foot, in the depression anterior to the junction of the first and second metatarsal bones
5	SP6	30 (50.85)	On the tibial aspect of the leg, 3-cun (finger-cun measurement) superior to the prominence of the medial malleolus, posterior to the medial border of the tibia.
6	EX-LE10	18 (30.51)	On the dorsum of foot, proximal to the web margins between all five toes, ai the dorso-ventral boundary. Both feet have a total of 8 points.
7	TE5	17 (28.81)	On the dorsal side of the forearm, on the line connecting Yangchi SJ-4 with the tip of the elbow, 2 cun above the transverse crease of the wrist between the ulna and the radius.
8	EX-UE9	15 (25.42)	On the flexor side of the forearm, proximal to the web margins between all five finger, at the junction of the red and white skin. Both hands altogether han
9	CV6	13 (22.03)	On the anterior median line of the lower abdomen, 1.5 cun below the navel.
10	GB34	12 (18.64)	On the lateral side of the lower leg, in the depression anterior and inferior to the
11	PC6	11 (18.64)	On the dorsum of the hand, between the second and third metacarpal bones, 0.5 cun posterior to the metacarpophalangeal joint.
12	SP10	9 (15.25)	On the prominence of the medial head of the quadriceps femoris, 2 cun above the medio-superior border of the base of patella.
13	SP9	9 (15.25)	On the medial side of the shank, at the sunken site posterior and inferior to the medial condyle of the tibia.
14	CV4	7 (11.86)	On the anterior median line of the lower abdomen, 3 cun below the navel.
15	PC7	7 (11.86)	In the middle of the transverse crease of the wrist, between the tendons of m.palmaris longus and m.flexor carpi radials.
16	SI3	7 (11.86)	On the ulna side of the palm, proximate to the fifth metacarpophalangeal joint, at the end of the transverse crease of the metacarpophalangeal joint, at the dorsoventral boundary.
17	ST41	7 (11.86)	At the sunken site which is on the midpoint of transverse crease that is on the border of the shank and the dorsum of the foot, between the tendon of the extensor hallucis longus and the tendon of the extensor digitorum longus.

#### 3.5.3 Association rules analysis

**Figure 14:**
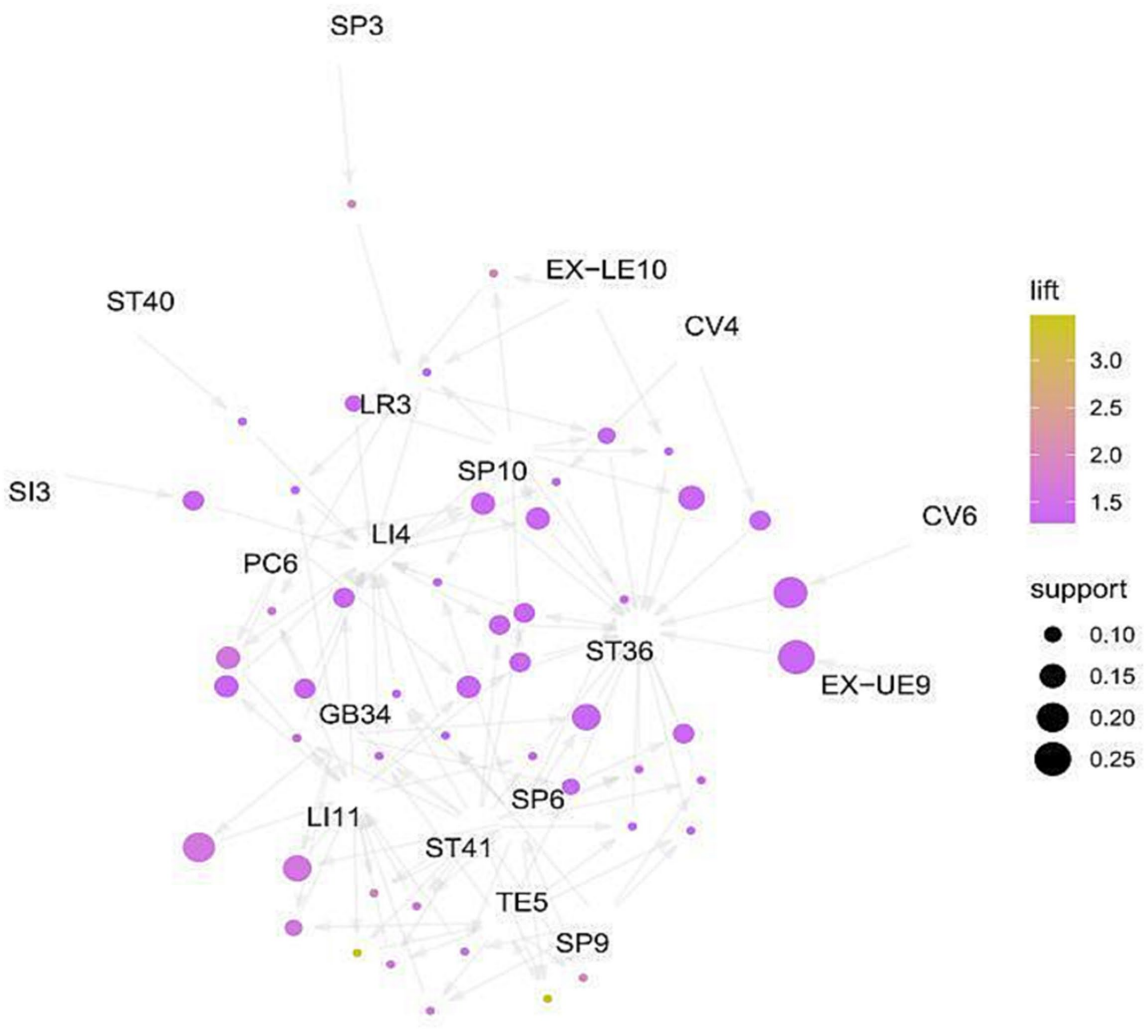
Networks.

The high-frequency acupoints were introduced into R4.3.1 software, and the Apriori algorithm was used to analyze the association rules. In Rstudio, the model is constructed using the Apriori algorithm with the minimum support level set to 0.3 and the minimum confidence level set to 0.7 for association analysis, 725 rules are obtained, among which the top 5 associations of support are {SP6}= > {ST36}, {LI4, SP6}= > {ST36}, {LI11, SP6}= > {ST36}, {LI11, LI4, SP6}= > {ST36}, {LI11, LI4, SP6} = > {ST36}, as shown in Table 4. The network graph is shown in Figure 14, where the size and color depth of the nodes represent the degree of support and enhancement, respectively, and the arrows indicate the correlation between the leading and trailing terms. The results show that ST36, LI4, LI11, SP6, and LR3 are the core acupoints for the treatment of this disease.

**Table 4:** Association rules analysis of common acupoints.

Preceding paragraph	Behind paragraph	Support %	Confidence %	Lift
{SP6}	{ST36}	0.49	0.97	1.27
{LI4,SP6}	{ST36}	0.42	1.00	1.31
{LI11,SP6}	{ST36}	0.41	1.00	1.31
{LI11,LI4,SP6}	{ST36}	0.37	1.00	1.31
{LI11,LR3}	{LI4}	0.36	0.95	1.22
{LI11,LR3}	{ST36}	0.36	0.95	1.25
{LI11,LI4,LR3}	{ST36}	0.34	0.95	1.25
{LI11,LR3,ST36}	{LI4}	0.34	0.95	1.22
{LI4,LR3,ST36}	{LI11}	0.34	0.95	1.52

## 4 Discussion

CIPN commonly occurs in the early stage of chemotherapy, and the typical clinical manifestations are symmetrical burning sensation, tingling sensation, anesthesia, numbness, and other symptoms in the “glove-sock” distribution (37–39). In addition, neurogenic pain is also one of the common manifestations of CIPN, which is more likely to cause physical and mental impact on patients than physical pain, and usually requires analgesic and antidepressant treatment (40). CIPN symptoms can be relieved after discontinuing the drug and can last for months or even years.

This study included 21 RCTs to evaluate the clinical efficacy and safety of acupuncture in the treatment of CIPN. The results of the meta-analysis showed that acupuncture was superior to conventional treatment in improving pain intensity, the intensity of the CIPN, MCV of the median nerves, SCV of the bilateral median, SCV of tibial nerve, SCV of sural nerves, quality of life score, physical functioning scale, and other outcome indicators. It is suggested that acupuncture is a safe, effective, simple and feasible non-drug therapy, which can be recommended for patients with CIPN.

Different acupuncture intervention variables may be important factors affecting the therapeutic effect. To conduct a rigorous examination of the efficacy of acupuncture, we embarked on a subgroup analysis based on acupuncture intervention variables. This endeavor aimed to empirically ascertain whether variations in acupuncture intervention variables have a significant impact on the robustness and consistency of the observed outcomes (16). In the results of the subgroup meta-analysis, there was no significant difference in pain intensity reduction between preventive acupuncture and the control group. Considering the small number of included studies, this may be the reason for the insignificant difference. Meanwhile, Greenlee, H. reported that a slightly higher proportion of patients in the sham acupuncture group believed that they had received real acupuncture treatment (35). Studies have shown that when people have a high expectation of a response to acupuncture treatment, this expectation may enhance treatment outcomes for patients who receive sham acupuncture. However, this expectation does not necessarily lead to the same results for patients who receive acupuncture. In terms of treatment duration, acupuncture was more effective in improving pain intensity at 0–6 weeks and 6–12 weeks, while there was no statistically significant difference above 12 weeks. The possible explanation is that the effector substance gradually diminishes as the course of treatment is prolonged, and the pain relief effect is not sustainable. The results of a meta-analysis of the frequency of acupuncture interventions showed that acupuncture therapy performed two to three times per week showed a more positive effect on pain relief. However, the number of included studies is limited, and the credibility of this result needs to be further verified in more multicenter, large-sample studies. In addition, it is important to note that the current research field lacks studies on interventions more than three times per week, which prevents us from directly inferring a clear relationship between intervention frequency and intervention effectiveness. Therefore, because current evidence only recommends acupuncture therapy two to three times per week, participants are not encouraged to improve CIPN neuropathic pain by increasing the frequency of intervention.

For safety assessment, 16 cases of hematoma, 1 case of needle stagnation, and 16 cases of pain were reported in the included studies. Subcutaneous hematoma and pain are related to acupuncture manipulation and can be relieved by themselves. Stagnation of the needle mainly refers to the phenomenon in which, after acupuncture or needle retention, the doctor feels that the needle is astringent, difficult to twist, lift, and insert, and comes out of the needle with severe pain to the patient. Most of these are due to mental strain on the patient or errors in needle handling by the doctor, which causes the muscle to twist the needle handle too much in a single direction. If the patient is overstrained, causing excessive contraction of the local muscles, the needle may be slightly extended, either in the vicinity of the acupuncture point to press or stretch the needle handle or in the vicinity of the additional needle to disperse the qi and blood and relieve the muscle tension. If the sticking of the needle is caused by the wrong needling manipulation or the single direction twist, the needle can be twisted back in the opposite direction, and the needle can be eliminated by scraping and plucking the needle handle to make the entangled tendon playback.

Acupuncture as a long-established means of TCM treatment, has been widely used in all kinds of diseases across the globe (41). Acupuncture therapy modulates the whole body through multiple systems and multiple targets (42). Previous studies have not only confirmed the remarkable effect of acupuncture in relieving neuropathic pain, but also further explored the molecular mechanisms behind it (43–45). Voltage-gated ion channels such as Na + and Ca + play a pivotal role in maintaining many key physiological processes in organisms, including excitability regulation of neurons, information transmission between synapses, muscle contraction, and hormone secretion, which are closely related to the occurrence and development of CIPN (46). Ren conducted electroacupuncture intervention on the PC6, SJ5S, SP6, and ST36 points in neuropathic rats, and the results showed that the expression level of Na + channels in the brain of successfully modeled rats increased, and electroacupuncture intervention could significantly reduce the level of Na + channels in the brain of rats (47). In terms of neurotransmitters, high extracellular glutamate concentrations may trigger excitotoxicity and lead to neuron death. Carla found that intraperitoneal injection of the mGluR5 antagonist MPEP could reverse the SNCV induced by bortezomib treatment in rats by lowering the concentration of Glu in the cerebrospinal fluid of rats. TRPV1 (Transient Receptor Potential Vanilloid 1) is a non-selective cation channel that is mainly expressed at the end of sensory neurons, particularly in neurons that are involved in the perception of pain and heat (48). TRPV1 can be activated by a variety of physical and chemical stimuli, the most well-known of which are high temperatures and capsaicin (the active ingredient in chili peppers). In recent years, more and more research has shown that TRPV1 is closely related to CIPN. Electrical acupuncture can inhibit the TLR4 (Toll-like receptor 4) signal transduction in DRG (dorsal root ganglion) neurons and simultaneously upregulate the expression of TRPV1 (transient receptor potential vanilloid type 1 subfamily member 1). These effects further result in the inhibition of activation of spinal glial cells, thereby alleviating the symptoms of CIPN (49). Xiao-Chen Li′s research indicates that acupuncture can inhibit the activation of M1 microglia and pro-inflammatory cytokines such as IL-1 and iNOS in the spinal cord of mice with CIPN induced by cisplatin through the suppression of spinal cord neuron miR-124 (50). GRK2, a G protein-coupled receptor kinase in the spinal cord, is involved in neuropathic pain and sensory abnormalities induced by cisplatin, and preventive electroacupuncture can increase GRK2 expression, thereby inhibiting the activation of microglia (51).

The data mining results show that LI4, ST36, LI11, LR3, and SP6 are the high-frequency acupoints for treating CIPN. (SP6 + ZST36), (LI4, SP6+ ST36), (LI11, SP6+ ST36), (LI11, LI4, SP6+ ST36), (LI11, LI4, SP6+ ST36) are the most commonly used combinations for this disease. According to traditional Chinese medicine theory, Hegu (LI4) is the yuan-source point of the hand Yangming meridian, which can treat finger numbness and paralysis of the upper limbs by promoting blood circulation and relieving pain. Zusanli (ST36) is the acupoint of the stomach meridian, which is the most important health acupoint in the human body and could promote the recovery QI of the spleen and stomach, promote the recovery and circulation of blood and Qi. There is the tibial nerve passing below it, so acupuncturing it can effectively improve the state of hypoxia and ischemia of the tibial nerve, stimulate the excitability of the nerve, and play a local acupoint treatment role. Quchi (LI11) is the he-sea point of the hand Yangming Meridian, which has the function of clearing heat dispersing wind, and regulating the circulation of Qi and blood. Quchi meridian deep layer is the radial nerve, which can also regulate the function of important nerves. SanYinjiao (SP6) is the rendezvous point of the Liver, Spleen, and Kidney meridians, which can improve the physiological state of the internal organs. The superficial layer below it is the branch of the saphenous nerve, and the deep layer has the tibial nerve, the posterior tibial artery, and the vein, which can regulate the function of the tibial nerve (52). Tai Chong (LR3) is a shu-stream acupoint and jing-river acupoint of the foot JueYin Liver Meridian, “liver controlling conveyance and dispersion, liver storing blood.” It is responsible for pain caused by the disrupted Qi flow, so Tai Chong has the function of regulating the flow of Qi. It is located on the sole of the foot, which has a local treatment effect.

### 4.1 Comparison of similar studies

At present, there are five meta-analyses on acupuncture treatment for CIPN (53–56). Consistent with previous systematic reviews and meta-analyses, acupuncture was associated with a significant reduction in pain intensity in patients with chemotherapy-associated peripheral neuropathy. However, this meta-analysis shows that acupuncture can significantly improve motor conduction velocities of nerve and sensory conduction velocities of nerve, which is different from previous evaluation results. This study evaluated the impact of acupuncture on the QOL of patients with CIPN. The assessment of QOL is an appropriate endeavor, constituting a multidimensional concept that comprehensively measures an individual’s satisfaction with their life (57). At the same time, a variety of intervention variables were used for acupuncture therapy in these meta-analyses, including but not limited to intervention duration, type of intervention, weekly intervention frequency, and total intervention duration. This inconsistency of intervention variables directly leads to significant differences in intervention effects among various studies, which further leads to the lack of uniformity and comparability in the formulation of clinical protocols, which has aroused widespread concern and deep concern among clinicians. Therefore, it is necessary to conduct a meta-analysis on the effects of acupuncture intervention with different intervention variables on CIPN, in order to provide evidence-based evidence for effective improvement of CIPN. In addition, this study follows the current guidelines for systematic review reports. It employs a comprehensive search strategy to retrieve the latest and high-quality randomized controlled trial evidence from multiple databases. The present study provides an updated synthesis of the current evidence of acupuncture in the treatment of CIPN.

### 4.2 Limitations and future research

The number of researches included in this paper is limited, and some studies have a high risk of bias, including unclear allocation hiding, inadequate blind method setting, and lack of Intention to treat analysis. Chemotherapy regimen, cancer type, cancer stage, and acupuncture treatment regimen may be the source of heterogeneity. In addition, the tools used to evaluate CNPI are not uniform and are mostly subjective scales. The study included only studies published in English and Chinese, which could be biased. In addition, in the process of diagnosing peripheral neuropathy, electromyography, and nerve conduction velocity testing are used as key tools for differentiating different types of nerve fibers. In particular, nerve conduction studies (NCS) have shown their unique applicability in detecting large fiber neuropathy. However, the detection results often show normal status in the face of CIPN (58). Considering the complexity of the underlying mechanisms of CIPN and the significant inconsistency between subjective symptoms and neurophysiological tests, there has been no established and widely accepted diagnostic method for CIPN so far.

## 5 Conclusion

Acupuncture can improve neuropathic pain, the intensity of the CIPN, nerve conduction velocity, quality of life, and other indicators in patients with CIPN, and has good safety. In addition, we observed that acupuncture intervention had a positive effect after the occurrence of CIPN symptoms, and all types of acupuncture intervention had a positive effect on the pain of patients with CIPN. At the same time, treatment intervention within 12 weeks could improve the pain intensity of patients with CIPN. ST36, LI4, LI11, SP6, and LR3 are core acupuncture points for CIPN treatment and are expected to be complementary therapies for CIPN.

## Data Availability

The original contributions presented in the study are included in the article/Supplementary material, further inquiries can be directed to the corresponding author.
