# Supplementary material for: Acupuncture in the treatment of chemotherapy-induced peripheral neuropathy: a meta-analysis and data mining
Source: Front Neurol. 2024 Oct 29;15:1442841. doi: 10.3389/fneur.2024.1442841 (PMC11565602; doi:10.3389/fneur.2024.1442841)
Supplement: Supplementary file 1 [file Data_Sheet_1.docx]

# 1 Search Strategy

**1.1 Pubmed**

| #1 | **Search: ((((chemotherapy-induced peripheral neuropathy[Title/Abstract]) OR (CNPN[Title/Abstract])) OR (chemotherapy-induced peripheral neurotoxicity[Title/Abstract])) OR (paclitaxel-induced peripheral neuropathy[Title/Abstract])) OR (oxaliplatin-induced peripheral neuropathy[Title/Abstract])** |
| --- | --- |
| #2 | **"Peripheral Nervous System Diseases/chemically induced"[Mesh]** |
| **#3** | **Search: ("Peripheral Nervous System Diseases/chemically induced"[Mesh]) OR (((((chemotherapy-induced peripheral neuropathy[Title/Abstract]) OR (CNPN[Title/Abstract])) OR (chemotherapy-induced peripheral neurotoxicity[Title/Abstract])) OR (paclitaxel-induced peripheral neuropathy[Title/Abstract])) OR (oxaliplatin-induced peripheral neuropathy[Title/Abstract]))** |
| #4 | **Search: (((((((((((acupuncture[MeSH Terms]) OR ("acupuncture analgesia"[MeSH Terms])) OR ("acupuncture therapy"[MeSH Terms])) OR ("acupuncture, ear"[MeSH Terms])) OR (auricular acupuncture[MeSH Terms])) OR (head acupuncture[MeSH Terms])) OR (fire acupuncture[MeSH Terms])) OR (manual acupuncture[MeSH Terms])) OR (electroacupuncture[MeSH Terms])) OR (meridians[MeSH Terms])) OR ("acupuncture points"[MeSH Terms])) OR ("trigger points"[MeSH Terms])** |
| #5 | **Search: (((((acupuncture*[Title/Abstract]) OR (electroacupuncture[Title/Abstract])) OR ("electro-acupuncture"[Title/Abstract])) OR (acupoint*[Title/Abstract])) OR (meridians[Title/Abstract])) OR (needling[Title/Abstract])** |
| **#6** | **(("Peripheral Nervous System Diseases/chemically induced"[Mesh]) OR (((((chemotherapy-induced peripheral neuropathy[Title/Abstract]) OR (CNPN[Title/Abstract])) OR (chemotherapy-induced peripheral neurotoxicity[Title/Abstract])) OR (paclitaxel-induced peripheral neuropathy[Title/Abstract])) OR (oxaliplatin-induced peripheral neuropathy[Title/Abstract]))) AND ((((((((acupuncture*[Title/Abstract]) OR (electroacupuncture[Title/Abstract])) OR ("electro-acupuncture"[Title/Abstract])) OR (acupoint*[Title/Abstract])) OR (meridians[Title/Abstract])) OR (needling[Title/Abstract])) OR ((((((((((((acupuncture[MeSH Terms]) OR ("acupuncture analgesia"[MeSH Terms])) OR ("acupuncture therapy"[MeSH Terms])) OR ("acupuncture, ear"[MeSH Terms])) OR (auricular acupuncture[MeSH Terms])) OR (head acupuncture[MeSH Terms])) OR (fire acupuncture[MeSH Terms])) OR (manual acupuncture[MeSH Terms])) OR (electroacupuncture[MeSH Terms])) OR (meridians[MeSH Terms])) OR ("acupuncture points"[MeSH Terms])) OR ("trigger points"[MeSH Terms]))) AND ((((chemotherapy-induced peripheral neuropathy[Title/Abstract]) OR (CNPN[Title/Abstract])) OR (chemotherapy-induced peripheral neurotoxicity[Title/Abstract])) OR (peripheral neuropathy[Title/Abstract])))** |

**1.2 Cochrane**

#1 (chemotherapy-induced peripheral neuropathy):ti,ab,kw OR (CNPN):ti,ab,kw OR (chemotherapy-induced peripheral neurotoxicity):ti,ab,kw OR (paclitaxel-induced peripheral neuropathy):ti,ab,kw OR (oxaliplatin-induced peripheral neuropathy):ti,ab,kw

#2 MeSH descriptor: [Acupuncture] explode all trees

#3 MeSH descriptor: [Acupuncture, Ear] explode all trees

#4 MeSH descriptor: [Electroacupuncture] explode all trees

#5 MeSH descriptor: [Meridians] explode all trees

#6 (acupuncture*):ti,ab,kw OR (electroacupuncture):ti,ab,kw OR ("electro-acupuncture"):ti,ab,kw OR (acupoint*):ti,ab,kw OR (meridians):ti,ab,kw

#7 (auriculotherap* or auriculoacupunct*):ti,ab,kw

#8 (needing):ti,ab,kw

#9 #2 OR #3 OR #4 OR #5 OR #6 OR #7 OR #8

#10 #1 AND #9 116

**1.3 WOS**

#1 (((((((((((((((ALL=(Acupuncture)) AND ALL=("acupuncture therapy")) OR ALL=("acupuncture analgesia")) OR ALL=("acupuncture, ear")) OR ALL=(auricular acupuncture)) OR ALL=(head acupuncture)) OR ALL=(fire acupuncture)) OR ALL=(manual acupuncture)) OR ALL=(Electroacupuncture)) OR ALL=("electro-acupuncture")) OR ALL=(Meridians)) OR ALL=("acupuncture points")) OR ALL=("trigger points")) OR ALL=(acupoint*)) OR ALL=(Needling)) OR ALL=(acupuncture*)

#2 ((((ALL=(chemotherapy-induced peripheral neuropathy)) OR ALL=(CNPN)) OR ALL=(chemotherapy-induced peripheral neurotoxicity)) OR ALL=(paclitaxel-induced peripheral neuropathy)) OR ALL=(oxaliplatin-induced peripheral neuropathy)

#3 #1 AND #2

**1.4 Embase**

**#1 'head acupuncture'**:ti,ab,kw OR **acupuncture**:ti,ab,kw OR **'acupuncture analgesia'**:ti,ab,kw OR **'acupuncture, ear'**:ti,ab,kw OR **'auricular acupuncture'**:ti,ab,kw OR **'fire acupuncture'**:ti,ab,kw OR **'manual acupuncture'**:ti,ab,kw OR **electroacupuncture**:ti,ab,kw OR **'electro-acupuncture'**:ti,ab,kw OR **meridians**:ti,ab,kw OR **'acupuncture points'**:ti,ab,kw OR **'trigger points'**:ti,ab,kw OR **acupoint***:ti,ab,kw OR **needling**:ti,ab,kw OR **acupuncture***:ti,ab,kw OR **auriculotherap***:ti,ab,kw OR **auriculoacupunct***:ti,ab,kw

**#2 cnpn**:ti,ab,kw OR **'chemotherapy-induced peripheral neuropathy'**:ti,ab,kw OR **'chemotherapy-induced peripheral neurotoxicity'**:ti,ab,kw OR **'paclitaxel-induced peripheral neuropathy'**:ti,ab,kw OR **'oxaliplatin-induced peripheral neuropathy'**:ti,ab,kw

#3 #1 AND#2

**1.5 CNKI**

(SU %= '化疗致周围神经损伤' OR SU %= '化疗所致周围神经损伤' OR SU %= '周围神经毒性' OR SU %= '周围神经损伤' OR SU %= '外周神经毒性' OR SU %= '化疗周围神经病变' OR SU %= '神经毒性' OR SU %= '化疗神经痛') AND (SU %= '针刺' OR SU %= '电针' OR SU %= '耳针' OR SU %= '头针' OR SU %= '穴位' OR SU %= '经脉'))

**1.6 WangFang**

主题:(化疗致周围神经损伤 or 化疗所致周围神经损伤or 周围神经毒性 or 周围神经损伤or 外周神经毒性or 化疗周围神经病变 or 神经毒性 or 化疗神经痛) and 主题:(针刺or 针灸 or 电针or 耳针or 穴位 or 温针灸 or 火针 or 穴位 or 经络)

**1.7 VIP**

M=(化疗致周围神经损伤 or 化疗所致周围神经损伤or 周围神经毒性 or 周围神经损伤or 外周神经毒性or 化疗周围神经病变 or 神经毒性 or 化疗神经痛) AND M=(针刺 or 电针 or 针灸or 耳针or 温针灸 or 火针 or 穴位 or 经络)

**1.8 CBM**

#1"周围神经系统疾病/化学诱导"[不加权:扩展]

#2"化疗致周围神经损伤"[常用字段:智能] OR "周围神经毒性"[常用字段:智能] OR "周围神经损伤"[常用字段:智能] OR "外周神经毒性"[常用字段:智能] OR "化疗周围神经病变"[常用字段:智能] OR "神经毒性"[常用字段:智能] OR "化疗神经痛"[常用字段:智能]

#3 #1OR#2

#4((((((("针灸疗法"[不加权:扩展]) OR "温针疗法"[不加权:扩展]) OR "电针疗法"[不加权:扩展]) OR "火针疗法"[不加权:扩展]) OR "耳针疗法"[不加权:扩展]) OR "头针疗法"[不加权:扩展]) OR "针刺穴位"[不加权:扩展]) OR "经络"[不加权:扩展]

#5 #4 AND #3
