# Supplementary material for: Acupuncture in the treatment of chemotherapy-induced peripheral neuropathy: a meta-analysis and data mining
Source: Front Neurol. 2024 Oct 29;15:1442841. doi: 10.3389/fneur.2024.1442841 (PMC11565602; doi:10.3389/fneur.2024.1442841)
Supplement: Supplementary file 2 [file Table_1.docx]

| **Section and Topic** | **Item #** | **Checklist item** | **Location where item is reported** |
| --- | --- | --- | --- |
| **TITLE** | | |  |
| Title | 1 | Acupuncture in the treatment of chemotherapy-induced peripheral neuropathy: a meta-analysis and data mining. |  |
| **ABSTRACT** | | |  |
| Abstract | 2 | Background: The efficacy and acupoint selection of acupuncture in treating chemotherapy-induced peripheral neuropathy (CIPN) remain controversial. This study aims to explore the specific efficacy and acupoint selection of acupuncture in treating CIPN through a meta-analysis and data mining.  Methods: Searching for clinical trials on acupuncture treatment for CIPN in 8 databases, evaluating its efficacy and safety through a meta-analysis, and exploring its acupoint selection through data mining.  Results: The meta-analysis included 21 studies and 2121 patients, showing that compared with the control group, the acupuncture group could significantly improve neuropathic pain intensity (SMD= -0.66, 95% CI [-1.07, -0.25], P =0.002), significantly reduce the NCI-CTCAE (SMD = -0.53, 95% CI [-0.75,-0.31], P < 0.001), significantly reduce the FACT-NXT score (SMD= 0.26, 95% CI [0.03,0.49], P < 0.05), significantly increase the motor conduction velocities (MCV) of median nerve (MD= 2.38, 95% CI [2.10, 2.67], P < 0.001), the sensory conduction velocities (SCV) of the median nerve (MD= 0.56, 95 %CI [-1.45, 2.57], P =0.58), the SCV of the tibial nerve (MD = 1.78, 95% CI [0.50, 3.05], P < 0.01), and the SCV of sural nerves (MD = 4.60, 95% CI [0.17, 9.02], P < 0.05), as well as improving the quality of life score (MD =7.35, 95% CI [1.53, 13.18], P =0.01). Data mining showed that the core acupoints for acupuncture treatment of CIPN were LI4, ST36, LI11, LR3, and SP6.  Conclusion: Acupuncture can improve the neuropathic pain intensity, the intensity of the CIPN, MCV of the median nerve, SCV of the tibial nerve and peroneal nerve, quality of life, and has good safety in CIPN patients. LI4 (Hegu), ST36 (Zusanli), LI11 (Quchi), LR3 (Taichong), and SP6 (Sanyinjiao) are the core acupuncture points for treating CIPN, and this protocol has the potential to become a supplementary treatment for CIPN. |  |
| **INTRODUCTION** | | |  |
| Rationale | 3 | However, the efficacy and point selection of acupuncture in the treatment of CIPN remain controversial (12), due to the lack of large-scale the clinical trials, and conflicting results have been reported in relevant randomized controlled trials. Recently, several high-level clinical studies were published. At present, randomized controlled trials of acupuncture intervention timing, course of treatment, intervention type, and treatment frequency are different, resulting in poor quality of evidence on the use of acupuncture for clinical treatment of patients with CIPN. |  |
| Objectives | 4 | This study will systematically evaluate the intervention effect of acupuncture on CIPN. This review differs from previous reviews in that it considers a wider range of outcome measures and analyses the effects of acupuncture intervention variables. At the time, explore the core acupoints of acupuncture on CIPN by using data mining technology, in order to provide reliable data for clinical applications. |  |
| **METHODS** | | |  |
| Eligibility criteria | 5 | (1) The study type was a randomized controlled clinical trial. (2) Participants were cancer patients with or without CPNI, regardless of cancer type or stage. (3) The intervention group was acupuncture/electroacupuncture or acupuncture/electroacupuncture combined with conventional rehabilitation/western medicine therapy. (4) The control group was treated with sham acupuncture or conventional rehabilitation or other Western medicine. (5) Primary outcome measures include any related measures of CPNI. |  |
| Information sources | 6 | Pubmed, Cochrane Library, Web of Science, Embase, China National Knowledge Infrastructure (CNKI), Wanfang Database, Weipu (VIP) Database, Sinomed (CBM), self-built repositories - published articles in January 2024. |  |
| Search strategy | 7 | 1.1 Pubmed  #1 Search: ((((chemotherapy-induced peripheral neuropathy[Title/Abstract]) OR (CNPN[Title/Abstract])) OR (chemotherapy-induced peripheral neurotoxicity[Title/Abstract])) OR (paclitaxel-induced peripheral neuropathy[Title/Abstract])) OR (oxaliplatin-induced peripheral neuropathy[Title/Abstract])  #2 "Peripheral Nervous System Diseases/chemically induced"[Mesh]  #3 Search: ("Peripheral Nervous System Diseases/chemically induced"[Mesh]) OR (((((chemotherapy-induced peripheral neuropathy[Title/Abstract]) OR (CNPN[Title/Abstract])) OR (chemotherapy-induced peripheral neurotoxicity[Title/Abstract])) OR (paclitaxel-induced peripheral neuropathy[Title/Abstract])) OR (oxaliplatin-induced peripheral neuropathy[Title/Abstract]))  #4 Search: (((((((((((acupuncture[MeSH Terms]) OR ("acupuncture analgesia"[MeSH Terms])) OR ("acupuncture therapy"[MeSH Terms])) OR ("acupuncture, ear"[MeSH Terms])) OR (auricular acupuncture[MeSH Terms])) OR (head acupuncture[MeSH Terms])) OR (fire acupuncture[MeSH Terms])) OR (manual acupuncture[MeSH Terms])) OR (electroacupuncture[MeSH Terms])) OR (meridians[MeSH Terms])) OR ("acupuncture points"[MeSH Terms])) OR ("trigger points"[MeSH Terms])  #5 Search: (((((acupuncture*[Title/Abstract]) OR (electroacupuncture[Title/Abstract])) OR ("electro-acupuncture"[Title/Abstract])) OR (acupoint*[Title/Abstract])) OR (meridians[Title/Abstract])) OR (needling[Title/Abstract])  #6 (("Peripheral Nervous System Diseases/chemically induced"[Mesh]) OR (((((chemotherapy-induced peripheral neuropathy[Title/Abstract]) OR (CNPN[Title/Abstract])) OR (chemotherapy-induced peripheral neurotoxicity[Title/Abstract])) OR (paclitaxel-induced peripheral neuropathy[Title/Abstract])) OR (oxaliplatin-induced peripheral neuropathy[Title/Abstract]))) AND ((((((((acupuncture*[Title/Abstract]) OR (electroacupuncture[Title/Abstract])) OR ("electro-acupuncture"[Title/Abstract])) OR (acupoint*[Title/Abstract])) OR (meridians[Title/Abstract])) OR (needling[Title/Abstract])) OR ((((((((((((acupuncture[MeSH Terms]) OR ("acupuncture analgesia"[MeSH Terms])) OR ("acupuncture therapy"[MeSH Terms])) OR ("acupuncture, ear"[MeSH Terms])) OR (auricular acupuncture[MeSH Terms])) OR (head acupuncture[MeSH Terms])) OR (fire acupuncture[MeSH Terms])) OR (manual acupuncture[MeSH Terms])) OR (electroacupuncture[MeSH Terms])) OR (meridians[MeSH Terms])) OR ("acupuncture points"[MeSH Terms])) OR ("trigger points"[MeSH Terms]))) AND ((((chemotherapy-induced peripheral neuropathy[Title/Abstract]) OR (CNPN[Title/Abstract])) OR (chemotherapy-induced peripheral neurotoxicity[Title/Abstract])) OR (peripheral neuropathy[Title/Abstract])))  1.2 Cochrane  #1 (chemotherapy-induced peripheral neuropathy):ti,ab,kw OR (CNPN):ti,ab,kw OR (chemotherapy-induced peripheral neurotoxicity):ti,ab,kw OR (paclitaxel-induced peripheral neuropathy):ti,ab,kw OR (oxaliplatin-induced peripheral neuropathy):ti,ab,kw  #2 MeSH descriptor: [Acupuncture] explode all trees  #3 MeSH descriptor: [Acupuncture, Ear] explode all trees  #4 MeSH descriptor: [Electroacupuncture] explode all trees  #5 MeSH descriptor: [Meridians] explode all trees  #6 (acupuncture*):ti,ab,kw OR (electroacupuncture):ti,ab,kw OR ("electro-acupuncture"):ti,ab,kw OR (acupoint*):ti,ab,kw OR (meridians):ti,ab,kw  #7 (auriculotherap* or auriculoacupunct*):ti,ab,kw  #8 (needing):ti,ab,kw  #9 #2 OR #3 OR #4 OR #5 OR #6 OR #7 OR #8  #10 #1 AND #9 116    1.3 WOS  #1 (((((((((((((((ALL=(Acupuncture)) AND ALL=("acupuncture therapy")) OR ALL=("acupuncture analgesia")) OR ALL=("acupuncture, ear")) OR ALL=(auricular acupuncture)) OR ALL=(head acupuncture)) OR ALL=(fire acupuncture)) OR ALL=(manual acupuncture)) OR ALL=(Electroacupuncture)) OR ALL=("electro-acupuncture")) OR ALL=(Meridians)) OR ALL=("acupuncture points")) OR ALL=("trigger points")) OR ALL=(acupoint*)) OR ALL=(Needling)) OR ALL=(acupuncture*)  #2 ((((ALL=(chemotherapy-induced peripheral neuropathy)) OR ALL=(CNPN)) OR ALL=(chemotherapy-induced peripheral neurotoxicity)) OR ALL=(paclitaxel-induced peripheral neuropathy)) OR ALL=(oxaliplatin-induced peripheral neuropathy)  #3 #1 AND #2  1.4 Embase  #1 'head acupuncture':ti,ab,kw OR acupuncture:ti,ab,kw OR 'acupuncture analgesia':ti,ab,kw OR 'acupuncture, ear':ti,ab,kw OR 'auricular acupuncture':ti,ab,kw OR 'fire acupuncture':ti,ab,kw OR 'manual acupuncture':ti,ab,kw OR electroacupuncture:ti,ab,kw OR 'electro-acupuncture':ti,ab,kw OR meridians:ti,ab,kw OR 'acupuncture points':ti,ab,kw OR 'trigger points':ti,ab,kw OR acupoint*:ti,ab,kw OR needling:ti,ab,kw OR acupuncture*:ti,ab,kw OR auriculotherap*:ti,ab,kw OR auriculoacupunct*:ti,ab,kw  #2 cnpn:ti,ab,kw OR 'chemotherapy-induced peripheral neuropathy':ti,ab,kw OR 'chemotherapy-induced peripheral neurotoxicity':ti,ab,kw OR 'paclitaxel-induced peripheral neuropathy':ti,ab,kw OR 'oxaliplatin-induced peripheral neuropathy':ti,ab,kw  #3 #1 AND#2  1.5 CNKI  (SU %= '化疗致周围神经损伤' OR SU %= '化疗所致周围神经损伤' OR SU %= '周围神经毒性' OR SU %= '周围神经损伤' OR SU %= '外周神经毒性' OR SU %= '化疗周围神经病变' OR SU %= '神经毒性' OR SU %= '化疗神经痛') AND (SU %= '针刺' OR SU %= '电针' OR SU %= '耳针' OR SU %= '头针' OR SU %= '穴位' OR SU %= '经脉'))  1.6 WangFang  主题:(化疗致周围神经损伤 or 化疗所致周围神经损伤or 周围神经毒性 or 周围神经损伤or 外周神经毒性or 化疗周围神经病变 or 神经毒性 or 化疗神经痛) and 主题:(针刺or 针灸 or 电针or 耳针or 穴位 or 温针灸 or 火针 or 穴位 or 经络)  1.7 VIP  M=(化疗致周围神经损伤 or 化疗所致周围神经损伤or 周围神经毒性 or 周围神经损伤or 外周神经毒性or 化疗周围神经病变 or 神经毒性 or 化疗神经痛) AND M=(针刺 or 电针 or 针灸or 耳针or 温针灸 or 火针 or 穴位 or 经络)  1.8 CBM  #1"周围神经系统疾病/化学诱导"[不加权:扩展]  #2"化疗致周围神经损伤"[常用字段:智能] OR "周围神经毒性"[常用字段:智能] OR "周围神经损伤"[常用字段:智能] OR "外周神经毒性"[常用字段:智能] OR "化疗周围神经病变"[常用字段:智能] OR "神经毒性"[常用字段:智能] OR "化疗神经痛"[常用字段:智能]  #3 #1OR#2  #4((((((("针灸疗法"[不加权:扩展]) OR "温针疗法"[不加权:扩展]) OR "电针疗法"[不加权:扩展]) OR "火针疗法"[不加权:扩展]) OR "耳针疗法"[不加权:扩展]) OR "头针疗法"[不加权:扩展]) OR "针刺穴位"[不加权:扩展]) OR "经络"[不加权:扩展]  #5 #4 AND #3 |  |
| Selection process | 8 | Two researchers (LLM and HYX) independently conducted literature screening and data extraction. Using Endnote 20.1 software, they removed duplicate articles. Following that, they screened out studies that did not meet the criteria by reading the titles and abstracts. Finally, they read the full texts of the remaining articles, determined the final selection based on the inclusion and exclusion criteria, and then extracted data from randomized controlled trials. |  |
| Data collection process | 9 | Specify the methods used to collect data from reports, including how many reviewers collected data from each report, whether they worked independently, any processes for obtaining or confirming data from study investigators, and if applicable, details of automation tools used in the process. |  |
| Data items | 10a | Primary outcome measures include any related measures of CPNI. |  |
|  | 10b | First author, publication year, study subjects, study design (randomized, blind), intervention measures, intervention period, evaluation measures, outcomes, and adverse events were included. Subgroups were split if there were three or multi-arm studies. |  |
| Study risk of bias assessment | 11 | The risk of bias in the included studies was assessed by two investigators (LLM and ACF) using the Cochrane Manual of Systematic Review 5.1.0 RCT Bias Risk Assessment tool, encompassing seven aspects: random sequence generation, allocation concealment, blinding of patients and investigators, blinding of outcome evaluators, incomplete outcome data reporting, selective reporting, and other sources of bias. In cases where there was disagreement between the two evaluators, a third evaluator (LHN) was involved to resolve any discrepancies. |  |
| Effect measures | 12 | Means and standard deviations of pretreatment and posttreatment results were collected for each group. Multiple levels of data are combined as continuous variables. Continuity variables were expressed in weighted mean difference (MD) or standardized mean difference (SMD), and 95% confidence intervals (CI) were calculated. |  |
| Synthesis methods | 13a | A total of 21 clinical studies were included, of which 3 were published in Chinese and 18 in English. The studies were published between 2013 and 2023, with 17 (80.95%) published in the last five years. Of the included studies, 10 studies were conducted in China, 5 studies in the United States, 1 study in collaboration with institutions in China and Iran, 1 in Brazil, 1 in Sweden, and 1 in Germany. A total of 2121 subjects were included, including 1048 in the experimental group and 1073 in the control group. Baseline information such as sex, age, cancer type, and chemotherapy regimen were comparable between the experimental and control groups. The basic characteristics of the included studies are shown in Table 1. |  |
|  | 13b | Means and standard deviations of pretreatment and posttreatment results were collected for each group. Multiple levels of data are combined as continuous variables. Continuity variables were expressed in weighted mean difference (MD) or standardized mean difference (SMD), and 95% confidence intervals (CI) were calculated. Referring to the study by Chen HT, for studies with two or more interventions/controls, participants were divided into different subgroups for assessment to prevent sample size overlap . If data is missing, the author will be contacted via email for further information. |  |
|  | 13c | The meta-analysis was performed using Revman 5.3 software |  |
|  | 13d | Describe any methods used to synthesize results and provide a rationale for the choice(s). If meta-analysis was performed, describe the model(s), method(s) to identify the presence and extent of statistical heterogeneity, and software package(s) used. |  |
|  | 13e | The meta-analysis was performed using Revman 5.3 software. The heterogeneity among the included studies was analyzed by χ2 test (α=0.05), and the heterogeneity was quantitatively determined by I2. When I2=0%, no heterogeneity was considered, and the fixed effect model was used to combine the data. When I2 was less than 50%, the heterogeneity was not considered significant, and the random effects model was conservatively used to combine the data. When I2≥50%, the heterogeneity is considered significant, and the random effects model is used to combine the data. |  |
|  | 13f | none |  |
| Reporting bias assessment | 14 | If more than 10 studies were included, the Begg’s tests and funnel plot were analyzed using Stata 17 software. If the funnel plot is symmetrical and P<0.05,  then the possibility of publication bias is relatively low. |  |
| Certainty assessment | 15 | The risk of bias in the included studies was assessed by two investigators (LLM and ACF) using the Cochrane Manual of Systematic Review 5.1.0 RCT Bias Risk Assessment tool, encompassing seven aspects: random sequence generation, allocation concealment, blinding of patients and investigators, blinding of outcome evaluators, incomplete outcome data reporting, selective reporting, and other sources of bias. In cases where there was disagreement between the two evaluators, a third evaluator (LHN) was involved to resolve any discrepancies. |  |
| **RESULTS** | | |  |
| Study selection | 16a | The article screening flow chart is in Figure 1. |  |
|  | 16b | A total of 2952 literature were searched, among which 1352 duplicate literature were excluded, 1418 irrelevant literature were excluded by title and abstract, 160 literature were excluded by full-text review, and 21 studies (12, 19-38) were finally included. |  |
| Study characteristics | 17 | A total of 21 clinical studies were included, of which 3 were published in Chinese and 18 in English. The studies were published between 2013 and 2023, with 17 (80.95%) published in the last five years. Of the included studies, 10 studies were conducted in China, 5 studies in the United States, 1 study in collaboration with institutions in China and Iran, 1 in Brazil, 1 in Sweden, and 1 in Germany. A total of 2121 subjects were included, including 1048 in the experimental group and 1073 in the control group. Baseline information such as sex, age, cancer type, and chemotherapy regimen were comparable between the experimental and control groups. The basic characteristics of the included studies are shown in Table 1. |  |
| Risk of bias in studies | 18 | The risk of random method bias was not clear in 1 study, and 10 studies describing envelopes and other ways to assign hiding were rated as low risk. Due to the special nature of acupuncture, 14 studies did not implement blind interventions for patients and participants rated as high risk. The risk of other bias was low. The details are shown in Figure 2. |  |
| Results of individual studies | 19 | For all outcomes, present, for each study: (a) summary statistics for each group (where appropriate) and (b) an effect estimate and its precision (e.g. confidence/credible interval), ideally using structured tables or plots. |  |
| Results of syntheses | 20a | The risk of random method bias was not clear in 1 study, and 10 studies describing envelopes and other ways to assign hiding were rated as low risk. Due to the special nature of acupuncture, 14 studies did not implement blind interventions for patients and participants rated as high risk. The risk of other bias was low. The details are shown in Figure 2. |  |
|  | 20b | Sixteen (12,20-22,26-28,30,32-37) studies evaluated peripheral neuropathic pain intensity involving a total of 824 patients. Of these, 4 studies (20,33,35,36) were evaluated using the visual analogue scale (VAS), 5 studies (12,26,30,34,37) using BPI-SF, and 4 studies (21,22,27,32) using the Numeric Rating Scale (NRS), 1 study (28) using EORTC QLQ-30-Pain scale, The results of heterogeneity analysis show p< 0.001 and I2 = 86%, indicating a high heterogeneity, so a random effects model was used for the analysis. The meta-analysis results showed that compared with the control group, the pain intensity in the acupuncture group decreased more (SMD= -0.66, 95% CI [-1.07, -0.25], P =0.002), illustrated in Figure 3. Sensitivity analysis showed that after removal of a study (36), I2 decreased to 48% (SMD= -0.43, 95% CI [-0.58, -0.28], P < 0.001) (see Figure 4). The intervention period of this study was greatly larger than that of other studies (3 chemotherapy cycles), suggesting that this study may be the source of high heterogeneity, and the length of acupuncture intervention affects the difference in acupuncture treatment results.  3.4.2 NCI-CTCAE  It into five studies (24,26,27,31,38), involving 322 patients, 160 in the treatment group and 162 people in the control group. The results of heterogeneity analysis show p =0.23 and I2 =28%, indicating homogeneity among the statistical variables. Therefore, a fixed effects model was used for analysis. Meta-analysis showed a statistically significant difference in the grade of peripheral neurotoxicity between the treatment and control groups (SMD = -0.53, 95% CI [-0.75,-0.31], P < 0.001), as shown in Figure 5.  3.4.3 FACT-NXT  A total of six studies (21,22,29,30,34,37) were included, involving 297 patients, of which 151 were in the treatment group and 146 were in the control group. The results of heterogeneity analysis show p = 0.09 and I2 =47%, indicating moderate homogeneity among the statistical variables. Therefore, a fixed effects model was used for analysis. Meta-analysis showed statistically significant differences in FACT-NXT scores between the treatment and control groups (SMD= 0.26, 95% CI [0.03,0.49], P < 0.05), as detailed in Figure 6.  3.4.4 Neural conduction velocity  (1) Motor conduction velocities (MCV) of median nerves  In total, three studies were included, involving 178 patients(21,23,36), with 88 patients in the treatment group and 90 in the control group. The results of heterogeneity analysis show p = 0.28 and I2 =21%, indicating homogeneity among the statistical variables. A fixed effects model analysis showed that there was a statistically significant difference in the motor conduction velocity of the median nerve between the treatment group and the control group (MD= 2.38, 95% CI [2.10, 2.67], P < 0.001), illustrated in Figure 7.  (2) Sensory conduction velocities (SCV) of the median nerve  There were three studies included in the analysis, involving a total of 178 patients(21,23,36), of which 88 were in the treatment group and 90 were in the control group. The results of heterogeneity analysis show p = 0.07 and I2 =63%, indicating a high heterogeneity. The random effects model analysis showed no statistically significant difference between the treatment group and the control group in median nerve conduction velocity (MD= 0.56, 95 %CI [-1.45, 2.57], P =0.58), as detailed in Figure 8.  (3) SCV of the tibial nerve  In a total of three studies involving 124 patients (21,27,31), with 62 patients in the treatment group and 62 in the control group. The results of heterogeneity analysis show p = 0.72 and I2 =0%, indicating homogeneity among various statistics. A fixed effects model analysis showed that there was a statistically significant difference in the median nerve motor conduction velocity between the treatment group and the control group (MD = 1.78, 95% CI [0.50, 3.05], P < 0.01), These findings are presented in Figure 9.  (4) SCV of the sural nerves  A total of 2 studies were included (27,36) involving 153 patients , with 76 in the treatment group and 77 in the control group. The results of heterogeneity analysis show p = 0.07 and I2 =69%, indicating a high heterogeneity. The random effects model analysis showed that there was a statistically significant difference in the median nerve motor conduction velocity between the treatment group and the control group (MD = 4.60, 95% CI [0.17, 9.02], P < 0.05), as shown in Figure 10.  3.4.5 Quality of Life Score (QOL)  Two studies (26,33) reported the total score of the EORTC QLQ-30. The results of heterogeneity analysis show p = 0.18 and I2 =44%, indicating moderate heterogeneity. The fixed effects model analysis showed a statistically significant difference in scores between the treatment and control groups (MD =7.35, 95% CI [1.53, 13.18], P =0.01), detailed in Figure 11. |  |
|  | 20c | Sensitivity analysis showed that after removal of a study (36), I2 decreased to 48% (SMD= -0.43, 95% CI [-0.58, -0.28], P < 0.001) (see Figure 4). The intervention period of this study was greatly larger than that of other studies (3 chemotherapy cycles), suggesting that this study may be the source of high heterogeneity, and the length of acupuncture intervention affects the difference in acupuncture treatment results. |  |
|  | 20d | none |  |
| Reporting biases | 21 | none |  |
| Certainty of evidence | 22 | Present assessments of certainty (or confidence) in the body of evidence for each outcome assessed. |  |
| **DISCUSSION** | | |  |
| Discussion | 23a | This study included 21 RCTs to evaluate the clinical efficacy and safety of acupuncture in the treatment of CIPN. The results of the meta-analysis showed that acupuncture was superior to conventional treatment in improving pain intensity, the intensity of the CIPN, MCV of the median nerves, SCV of the bilateral median, SCV of tibial nerve, SCV of sural nerves, quality of life score, physical functioning scale, and other outcome indicators. It is suggested that acupuncture is a safe, effective, simple and feasible non-drug therapy, which can be recommended for patients with CIPN. |  |
|  | 23b | The number of researches included in this paper is limited, and some studies have a high risk of bias, including unclear allocation hiding, inadequate blind method setting, and lack of Intention to treat analysis. Chemotherapy regimen, cancer type, cancer stage, and acupuncture treatment regimen may be the source of heterogeneity. |  |
|  | 23c | The study included only studies published in English and Chinese, which could be biased. |  |
|  | 23d | In addition, in the process of diagnosing peripheral neuropathy, electromyography, and nerve conduction velocity testing are used as key tools for differentiating different types of nerve fibers. In particular, nerve conduction studies (NCS) have shown their unique applicability in detecting large fiber neuropathy. However, the detection results often show normal status in the face of CIPN(60). Considering the complexity of the underlying mechanisms of CIPN and the significant inconsistency between subjective symptoms and neurophysiological tests, there has been no established and widely accepted diagnostic method for CIPN so far. |  |
| **OTHER INFORMATION** | | |  |
| Registration and protocol | 24a | The present study was conducted by the 2020 Statement on Preferred Reporting Items for Systematic Reviews and Meta-analyses (PRISMA 2020). This systematic review and meta-analysis has been registered with the PROSPERO Registry (CRD42022370952). |  |
|  | 24b | A protocol was not prepared. |  |
|  | 24c | None. |  |
| Support | 25 | This study was financially supported by the National Science Foundation of China (No. 82274674). |  |
| Competing interests | 26 | Competing interest: The authors declare that they have no competing interests. |  |
| Availability of data, code and other materials | 27 | Cochrane Risk of Bias Assessment Tool 2.0 (RoB2). The Cochrane Handbook for Systematic Reviews 5.1.0.  Review Manager (RevMan) [Computer program]. Version 5.3, The Cochrane Collaboration.  EndNote 20.1 Philadelphia: Clarivate Analytics. |  |

*From:*  Page MJ, McKenzie JE, Bossuyt PM, Boutron I, Hoffmann TC, Mulrow CD, et al. The PRISMA 2020 statement: an updated guideline for reporting systematic reviews. BMJ 2021;372:n71. doi: 10.1136/bmj.n71

For more information, visit: <http://www.prisma-statement.org/>
